# Unveiling the impact of DNA-methylation age acceleration on mortality risk in diabetes and pre-diabetes: insights from the US NHANES program

**DOI:** 10.1186/s13148-025-01886-0

**Published:** 2025-05-16

**Authors:** Shuang Wu, Ziyi Zhong, Yimeng Wang, Jingyang Wang, Siqi Lyu, Hongyu Liu, Yang Chen

**Affiliations:** 1 National Center for Cardiovascular Disease, Fuwai Hospital, Chinese Academy of Medical Sciences and Peking Union Medical College, Beijing, People’s Republic of China; 2 National Clinical Research Center of Cardiovascular Diseases, National Center for Cardiovascular Disease, Fuwai Hospital, Chinese Academy of Medical Sciences and Peking Union Medical College, Beijing, People’s Republic of China; 3 Liverpool Centre for Cardiovascular Science at University of Liverpool, Liverpool John Moores University and Liverpool Heart and Chest Hospital, William Henry Duncan Building, 6 West Derby Street, Liverpool, L7 8TX UK; 4 Department of Musculoskeletal Ageing and Science, Institute of Life Course and Medical Sciences, University of Liverpool, Liverpool, UK; 5 Department of Cardiovascular Medicine, The Second Affiliated Hospital, Jiangxi Medical College, Nanchang University, Nanchang, Jiangxi People’s Republic of China; 6 Department of Cardiovascular and Metabolic Medicine, Institute of Life Course and Medical Sciences, University of Liverpool, Liverpool, UK

**Keywords:** DNA-methylation age, DNA-methylation age acceleration, GrimAge2Mort, AgeAccelGrim2, Diabetes, Pre-diabetes, Mortality

## Abstract

**Background:**

Diabetes ranks as the ninth leading cause of death globally, and DNA-methylation age acceleration (DNAmAA) is closely linked to lifespan. However, the impact of DNAmAA on long-term outcomes in specific populations with diabetes and pre-diabetes has not yet been comprehensively studied.

**Methods:**

This retrospective cohort study utilized data from the National Health and Nutrition Examination Survey (NHANES) 1999–2002, including participants aged 20 years or older diagnosed with diabetes or pre-diabetes. DNAmAA was defined as the difference between epigenetic age and chronological age. Multiple generations of DNAmAA measures were included. Cox proportional hazards regression models were employed to estimate the associations between DNAmAAs and all-cause, cardiovascular, and non-cardiovascular mortality.

**Results:**

A total of 1,199 participants were included, with a mean age of 64.20 (0.46) years; 621 (51.8%) were male. Significant correlations were observed between chronological age and all DNA-methylation ages in both diabetes and pre-diabetes groups. Over a mean follow-up of 14.13 (5.90) years, 662 deaths were recorded. AgeAccelGrim2 exhibited the strongest association with mortality. Each 5-unit increase in AgeAccelGrim2 was associated with an elevated risk of all-cause mortality (HR 1.35, 95% CI 1.23–1.49), cardiovascular mortality (HR 1.50, 95% CI 1.25–1.80), and non-cardiovascular mortality (HR 1.30, 95% CI 1.16–1.46). These associations remained significant in participants with diabetes and pre-diabetes. Mediation analysis revealed that AgeAccelGrim2 significantly mediates the association between health-related exposures (including the Oxidative Balance Score, Life’s Simple 7 score, and frailty score) and all-cause mortality in diabetes and pre-diabetes populations.

**Conclusions:**

AgeAccelGrim2 could serve as a valuable biomarker for mortality risk specific to populations with diabetes and pre-diabetes, offering potential applications in personalized management strategies and risk stratification.

**Supplementary Information:**

The online version contains supplementary material available at 10.1186/s13148-025-01886-0.

## Introduction

Type 2 diabetes (T2D) is a metabolic disorder associated with aging, characterized by chronic hyperglycemia and insulin resistance and is one of the most prevalent and serious chronic diseases worldwide [1, 2]. Individuals with T2D face a significantly higher risk of mortality and complications, including cardiovascular disease, kidney failure, neurodegenerative disorders, retinopathy, and neuropathy [3, 4]. The global rise in diabetes prevalence, driven by urbanization, sedentary lifestyles, and aging populations, has created a substantial health and economic burden, particularly in low- and middle-income countries where healthcare resources are limited [5]. The pathogenesis of T2D is complex, and DNA methylation (DNAm) may play a role in its development. Studies have shown that DNAm alterations occur in the pancreatic islets, liver, and skeletal muscle of individuals with diabetes, which disrupt gene expression and impair insulin secretion, ultimately leading to metabolic dysregulation [6]. 

DNAmAge, which estimates biological age based on predictable changes in DNAm patterns with age, has demonstrated strong correlations with morbidity, mortality, and a wide range of age-related diseases [7]. The evolution of biological clocks from first- to third-generation models marks significant progress: first-generation clocks (e.g., HorvathAge [8], HannumAge [9]) aim to predict chronological age; second-generation clocks (e.g., PhenoAge [10], ZhangAge [11]) estimate biological age using health biomarkers; third-generation clocks (e.g., GrimAge2Mort [12], DunedinPoAm [13]) aim to predict mortality, disease outcomes, and aging pace by integrating disease-specific biomarkers. DNAmAge acceleration (DNAmAA), defined as the discrepancy between biological age, as estimated by these clocks, and chronological age, is also linked to elevated risks of age-related diseases and mortality [14].

PhenoAge, developed by Levine et al. in 2018, predicts aging rates and health risks by analyzing DNAm patterns combined with clinical biomarkers such as inflammation, metabolism, and organ function indicators [10]. GrimAge, constructed based on seven DNAm-based plasma protein markers and pack-years of smoking, showing superior performance in predicting lifespan, healthspan and age-related conditions such as cardiovascular diseases and cognitive decline [15, 16]. GrimAgeV2, as its enhanced version by incorporating two additional DNAm-based estimators (logCRP and logA1C), further improves its predictive performance on mortality and age-related conditions [12]. DunedinPoAm, as a third-generation epigenetic clock, measures the pace of biological aging by analyzing DNAm patterns and offers insights into the rate of aging rather than just biological age [13].

In the field of diabetes, these epigenetic clocks have been extensively studied for their associations with disease onset, progression, and related complications. Multiple studies have shown that GrimAgeV2 is strongly linked to both the onset of diabetes and the development of diabetes-related complications [17–20]. Regarding mortality outcomes, Sabbatinelli et al. conducted a case–control study involving 50 individuals with T2D, revealing that elevated DNAmPhenoAge and accelerated DunedinPoAm were independently associated with increased mortality risk [21]. Similarly, Jiang et al. investigated the role of biological aging in cardiometabolic multimorbidity development and mortality among 341,159 UK Biobank participants, showing that PhenoAge was strongly associated with higher risks of progression from cardiometabolic disease to multimorbidity and mortality [22]. 

Despite these advancements, the predictive ability of DNAmAA measures for mortality in diabetes and pre-diabetes populations remains underexplored. In this longitudinal study, we focused on individuals with diabetes and pre-diabetes, comprehensively evaluating the impact of ten epigenetic clock accelerations, including AgeAccelGrim2 and DunedinPoAm, on long-term outcomes. Additionally, we investigated the potential mediating role of DNAmAA in the relationships between health-related exposures and mortality risk.

## Methods

### Study design and dataset generation

The data used in this study were derived from the 1999–2002 National Health and Nutrition Examination Survey (NHANES). NHANES is an ongoing national program overseen by the National Center for Health Statistics under the Centers for Disease Control and Prevention, focusing on US non-institutionalized civilians. NHANES utilizes a complex, multistage probability sampling design, integrating face-to-face interviews, physical examinations, and laboratory tests to collect data. For this study, we included 1,199 eligible participants. Detailed description of the inclusion/exclusion criteria is provided in eFigure 1.

NHANES received ethical approval from the National Center for Health Statistics Ethics Review Board, and all participants provided written informed consent. This study was followed the Strengthening the Reporting of Observational Studies in Epidemiology reporting guideline (eTable 1).

### Outcome definition

The primary outcome in this study was all-cause mortality. Secondary outcomes included cardiovascular and non-cardiovascular mortality. The date and cause of death were linked to National Death Index records through December 31st, 2019. We used the 10th revision of the International Classification of Diseases to determine the cause of death. The participants were followed from the date of their survey participation until death or the end of the follow-up period.

### Laboratory methodology

DNA was extracted from whole blood and stored at −80 °C. Bisulfite conversion was performed on 500 ng of DNA using the Zymo EZ DNA Methylation Kit (cat# D5001, Zymo Research, Irvine, CA, USA). Methylation profiling was conducted using the Illumina Infinium MethylationEPIC BeadChip v1.0 (cat# WG317-1001, Illumina, San Diego, CA, USA), following standard protocols for hybridization, amplification, and labeling. The raw methylation data underwent quality control procedures, including outlier detection, imputation, and normalization, and were subsequently used to generate epigenetic biomarkers based on established algorithms: HorvathAge [8], HannumAge [9], SkinBloodAge [23], Vidal-BraloAge [24], WeidnerAge [25], PhenoAge [10], ZhangAge [11], LinAge [26], GrimAge2Mort [12], and DunedinPoAm [13]. Additional details on the DNA-methylation data processing are available at https://wwwn.cdc.gov/nchs/nhanes/dnam/.

These DNAmAge algorithms can be categorized into first-generation, second-generation, and third-generation clocks, each with distinct methodologies, biological focuses, and applications. First-generation clocks (e.g., HorvathAge, HannumAge, SkinBloodAge) predict chronological age using DNAm patterns. Second-generation clocks (e.g., PhenoAge, ZhangAge, LinAge) incorporate health-related biomarkers to estimate biological age and assess disease risk. Third-generation clocks (e.g., GrimAge2Mort) integrate disease-related biomarkers and longitudinal data to predict mortality risk and the pace of biological aging (e.g., DunedinPoAm). These algorithms differ in their CpG site selection, which may reflect unique biological pathways or tissue-specific methylation patterns, and were trained on diverse datasets, ranging from multi-tissue samples (e.g., HorvathAge) to blood-specific samples (e.g., HannumAge) or longitudinal cohorts (e.g., DunedinPoAm). These differences in CpG site selection, training datasets, prediction targets, and biological focus collectively contribute to the variations observed among DNAmAge values. Detailed procedures for each DNAmAge algorithm are provided in the Supplemental eMethods. In this study, DNAmAA was calculated as the residuals from the regression of DNAmAge on chronological age, as described in the previous work [2, 7].

### Variables collection

In addition to variable related to DNAmAA, we collected a range of variables for analysis including chronological age, sex, body mass index (BMI), race and ethnicity, poverty-to-income ratio (PIR), smoking status, systolic blood pressure, diastolic blood pressure, systemic immune inflammation index (SII), Oxidative Balance Score (OBS), Life’s Simple 7 (LS7) score, frailty score, Geriatric Nutritional Risk Index (GNRI), comorbidities (atherosclerotic cardiovascular disease [ASCVD], hypertension, hypercholesterolemia, chronic kidney disease [CKD]), hemoglobin A1c, total cholesterol, high-density lipoprotein cholesterol, estimated glomerular filtration rate (eGFR), urine albumin-to-creatinine ratio (UACR).

Race and ethnicity were categorized as non-Hispanic White, non-Hispanic Black, Mexican American, Hispanic and other race (which included participants who identified as non-Hispanic multiracial). Race and ethnicity data were collected as a confounding factor to account for differences among racial and ethnic groups in susceptibility to diabetes, pre-diabetes, and related health outcomes. Therefore, controlling for race and ethnicity helps ensure that any observed associations between DNAmAA and mortality are not confounded by these factors.

SII, calculated as (platelet count × neutrophil count)/lymphocyte count, is a marker of systemic inflammation and immune response. It has been associated with increased risks of chronic diseases and mortality, making it a valuable prognostic tool [28]. OBS is a composite measure of pro-oxidant and antioxidant exposures, including dietary and lifestyle factors. Higher OBS values indicate a greater antioxidant capacity, which has been linked to reduced risks of oxidative stress-related conditions, such as cardiovascular disease and diabetes [29]. LS7, developed by the American Heart Association, is a composite score based on seven modifiable cardiovascular health factors, including smoking, diet, and physical activity. Higher LS7 scores are associated with better cardiovascular health and reduced risks of diabetes and mortality [30]. Frailty index was developed by Searle and colleagues and constructed using 40 variables associated with health status, covering multiple physiological systems, This approach generates a continuous score ranging from total fitness (0) to total frailty (1), providing a comprehensive measure of an individual’s vulnerability to adverse health outcomes [31]. GNRI is a nutritional assessment tool calculated from serum albumin levels, body weight, and height. It is widely used to evaluate malnutrition risk in older adults and has been associated with mortality, frailty, and other adverse health outcomes [32]. Detailed definitions of these covariates are provided in Supplemental eMethod.

### Statistical analysis

NHANES initially employed a complex survey design, with all results weighted to yield estimates that are nationally representative of the non-institutionalized civilian population of the USA. In this study, we used the ‘WTDN4YR’ weights from the NHANES 1999–2000 and 2001–2002 DNA-methylation array and epigenetic biomarkers dataset for analysis. Data analysis was conducted between August 1st and October 14th, 2024.

The extent of missing data for each variable is detailed in eTable 2, with PIR exhibiting the highest proportion of missing values at 11.59%, whereas other variables demonstrated missing rates between 0.1% and 0.5%. To address these missing data, we employed the ‘mice’ package in R for multiple imputation using chained equations. A total of 20 imputed datasets were generated through iterative imputation. For each variable, we selected the imputed dataset that minimized the relative difference between the imputed mean and the original mean of the incomplete data. These selected values were combined to construct a single complete dataset, which was used for all subsequent analyses. For the descriptive statistics, continuous variables are expressed as mean (standard deviation [SD]), and categorical variables are expressed as numbers and percentages. Pearson’s correlation analysis was performed to analyze the relationships between chronological age with each DNAmAge and each DNAmAA, and the correlation coefficients (r) were given. Additionally, the relationships between each DNAmAA with laboratory indicators (e.g., eGFR, UACR), multidimensional scores (e.g., SII, OBS), and vital signs were analyzed by partial correlation analysis adjusted by chronological age, sex, and smoking, and the r values were given.

We examined whether each DNAmAA and covariates met the proportional hazards assumption of the Cox proportional hazards model. We adjusted for confounders (including chronological age, sex, race and ethnicity, PIR, smoking status, BMI, GNRI, ASCVD, hypertension, hyperlipidemia, and CKD). These covariates were selected based on literature, clinical relevance, and our data characteristics [33]. Laboratory indicators (e.g., CRP, HbA1c) and multidimensional score (e.g., OBS, SII) were not included into models due to potential multicollinearity with DNAmAA. We reported hazard ratios (HR) with 95% confidence intervals (CI) for each 5-unit increase in the DNAmAAs and for 10% increases in the DunedinPoAm pace of aging to quantify the associations between each DNAmAA and mortality outcomes. For DNAmAAs significantly associated with all-cause mortality, these variables were also analyzed categorically in tertiles, with the first tertile serving as the reference for calculating HRs and 95% CIs. To further validate the robustness of our findings, we conducted the following sensitivity analyses. First, we adjusted for all variables listed in Table 1 in the Cox regression model to account for their potential influence on the association between each DNAmAA and mortality outcomes. Second, we excluded participants with missing data to minimize the impact of missing values on the results. Finally, we removed participants who died within the first two years of follow-up to reduce the risk of reverse causality.

**Table 1:** Baseline characteristics of all participants categorized by survival status

Characteristics	Overall participants *N* = 1199	Survivors *N* = 537	Non-survivors *N* = 662
Age, mean (SD), years	64.20 (0.46)	58.73 (0.41)	69.55 (0.53)
Male, n (%)	621 (51.79)	266 (47.40)	355 (46.45)
*Ethnicity, n (%)*			
Non-Hispanic White Non-Hispanic Black Mexican American Hispanic and Other	364 (30.36) 313 (26.11) 392 (32.69) 130 (10.84)	136 (67.26) 127 (11.98) 194 (5.06) 80 (15.69)	228 (73.62) 186 (13.10) 198 (4.35) 50 (8.93)
Body mass index, mean (SD), kg/m^2^	30.64 (0.28)	30.77 (0.45)	30.50 (0.36)
Systolic blood pressure, mean (SD), mmHg	135.20 (0.75)	132.14 (1.28)	138.17 (1.13)
Diastolic blood pressure, mean (SD), mmHg	71.53 (0.68)	75.89 (0.93)	67.19 (0.88)
Poverty income ratio, mean (SD)	2.85 (0.09)	3.22 (0.15)	2.47 (0.09)
*Smoking status, n (%)*			
Never smoker Former smoker Current smoker	575 (48.08) 434 (36.29) 187 (15.64)	272 (43.91) 188 (37.47) 75 (18.62)	303 (44.87) 246 (37.08) 112 (18.04)
*Comorbidities, n (%)*			
Cardiovascular disease	258 (21.52)	63 (11.17)	195 (33.60)
Hypertension	829 (69.14)	329 (60.43)	500 (74.03)
Hypercholesterolemia	1016 (84.74)	452 (87.61)	564 (88.63)
Chronic kidney disease	412 (34.86)	110 (20.23)	302 (42.16)
*Laboratory indicators*			
HDL-C, mean (SD), mmol/L	1.24 (0.01)	1.23 (0.02)	1.24 (0.02)
Total Cholesterol, mean (SD), mmol/L	5.55 (0.05)	5.60 (0.06)	5.51 (0.08)
eGFR, mean (SD), ml/min/1.73 m^2^	77.94 (0.83)	84.37 (0.95)	71.65 (1.10)
Hemoglobin A1c, mean (SD), %	6.36 (0.06)	6.09 (0.08)	6.63 (0.09)
UACR, mean (SD), mg/g	86.86 (16.71)	35.24 (8.04)	138.19 (30.71)
C-reactive protein, mean (SD), mg/dl	0.66 (0.06)	0.57 (0.07)	0.75 (0.10)
SII, mean (SD)	638.00 (22.71)	625.84 (38.78)	650.02 (26.41)
*Multidimensional score*			
Life’s Simple 7 score, mean (SD)	5.92 (0.11)	6.21 (0.13)	5.63 (0.12)
Frailty score, mean (SD)	0.19 (0.00)	0.16 (0.01)	0.21 (0.00)
Oxidative Balance Score, mean (SD)	17.27 (0.29)	18.23 (0.54)	16.32 (0.33)
Geriatric Nutritional Risk Index, mean (SD)	121.53 (0.52)	122.19 (0.76)	120.85 (0.75)

eGFR, estimated glomerular filtration rate; HDL-C, high-density lipoprotein cholesterol; SD, standard deviation; SII, Systemic immune inflammation index; UACR, urine albumin-to-creatinine ratio

Given the significance of AgeAccelGrim2 and DunedinPoAm in predicting mortality, we compared their predictive performance using DeLong’s test, evaluating the area under the curve of the receiver operating characteristic curves across overall participants, as well as subgroups with diabetes and pre-diabetes. Our results indicate that AgeAccelGrim2 shows a stronger predictive advantage over DunedinPoAm for all-cause mortality (eFigure 2). We conducted restricted cubic spline (RCS) analyses with three knots to examine potential nonlinear associations between AgeAccelGrim2 and mortality outcomes. Subgroup analyses were performed to evaluate the association between AgeAccelGrim2 and all-cause mortality, stratified by chronological age (≥ 65 and < 65 years), sex (male and female), and BMI (≥ 30 and < 30 kg/m^2^). We also evaluated the predictive effect of AgeAccelGrim2 on mortality in non-diabetic/non-pre-diabetic populations to compare whether differences exist relative to diabetic populations.

We investigated the potential mediating role of AgeAccelGrim2 in the association between health-related exposures (in diabetes population: HbA1c, eGFR, OBS, LS7, frailty; in pre-diabetes population: OBS, LS7, frailty) and all-cause mortality using the mediation package in R. As AgeAccelGrim2 serves as a biomarker of biological aging, reflecting accumulated physiological damage, its use as a mediator helps elucidate how health-related factors influence mortality risk by accelerating or decelerating epigenetic aging. Adjusted models were applied, and mediation analyses with 500 bootstrap resamples were conducted to estimate the direct and indirect effects of AgeAccelGrim2.

All analyses were conducted using R version 3.5.2 (R Project for Statistical Computing, Vienna, Austria). *P* values were two-tailed, and < 0.05 was considered statistically significant.

## Results

### Baseline characteristics

A total of 1,199 eligible patients were included, comprising 542 patients with diabetes and 657 patients with pre-diabetes (mean [SD] age: 64.20 (0.46) years; 621 males [51.8%]). During a mean follow-up period of 14.13 (5.90) years, 662 participants (55.2%) died (diabetes: 347 [64.0%]; pre-diabetes: 315 [47.9%]). In terms of race and ethnicity, 364 (30.36%) were non-Hispanic White, 313 (26.11%) were non-Hispanic Black, 392 (32.69%) were Mexican American, and 130 (10.84%) were other Hispanic or other race. The baseline characteristics of all participants are presented in Table 1; the details for those with diabetes and pre-diabetes are outlined in eTable 3 and eTable 4, respectively.

### Chronological age, DNAmAge, and DNAmAA

**Fig. 1:**
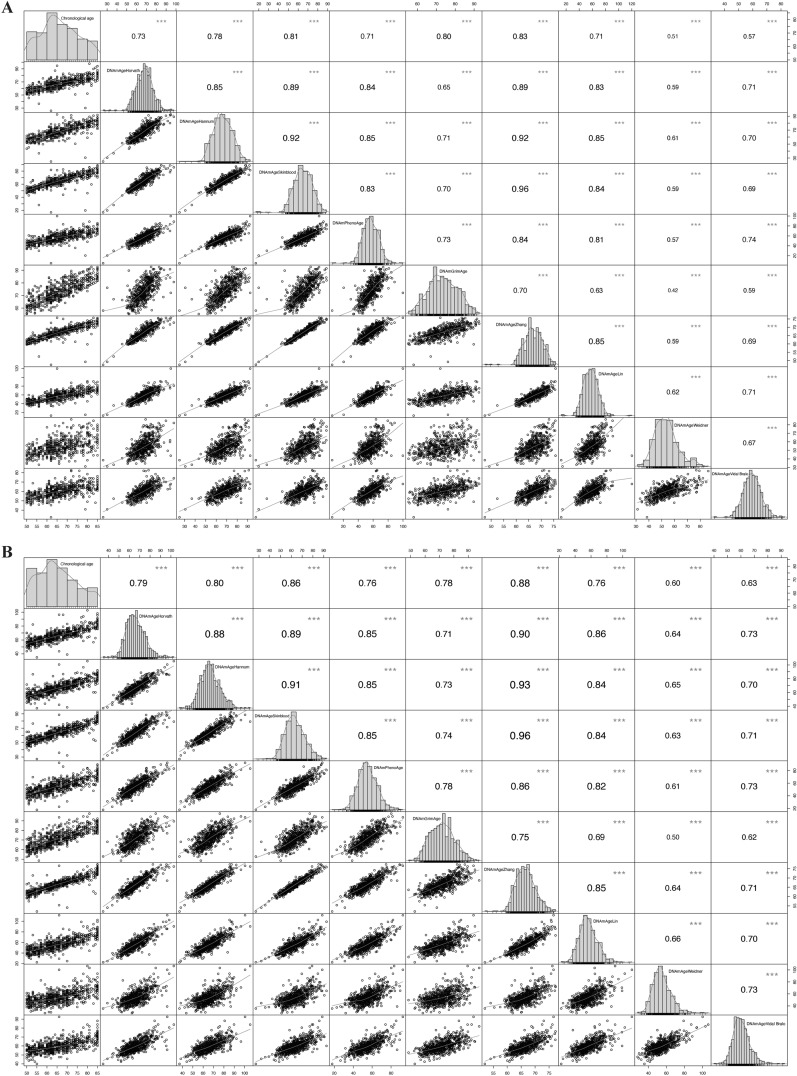
The correlation analyses between chronological age and all DNA-methylation ages. Participants with diabetes **A** and pre-diabetes **B**. * *P* < 0.05; ** *P* < 0.01; *** *P* < 0.001

eTable 5 shows the distribution of each DNAmAge and DNAmAA. Figure 1 presents the correlations between chronological age and all DNAmAges. In the diabetes population, significant correlations were found between chronological age and all DNAmAges, with r ranging from 0.51 to 0.92 (all *P* < 0.001). Among them, the correlation between Horvath DNAmAge and chronological age was the strongest (r = 0.92). Similarly, in the pre-diabetes population, significant relationships were observed between chronological age and each DNAmAge, with r ranging from 0.50 to 0.93 (all *P* < 0.001). In addition, the correlations among the various DNAmAAs were also significant (*P* < 0.001), as illustrated in eFigure 2.

### DNAmAAs with laboratory indicators, multidimensional scores, and vital signs

eFigure 3 presents the results of partial correlation analyses between different DNAmAAs and multiple variables. For both diabetes and pre-diabetes populations, the correlations between DNAmAAs and laboratory indicators, multidimensional scores, and vital signs varied. In the diabetes population, SII showed a significant positive correlation with multiple DNAmAAs, particularly with DunedinPoAm (r = 0.311, *P* < 0.001) and Vidal-BraloAA (r = 0.296, *P* < 0.001). Conversely, the OBS was significantly negatively correlated with AgeAccelGrim2 (r = −0.086, *P* < 0.05). The frailty score and UACR demonstrated positive correlations with AgeAccelGrim2, with r of 0.171 and 0.135, respectively. In the pre-diabetes population, SII was also correlated with various DNAmAAs, especially with Vidal-BraloAA (r = 0.280, *P* < 0.001) and LinAA (r = 0.209, *P* < 0.001). Additionally, AgeAccelGrim2 was correlated with the OBS (r = −0.103, *P* < 0.01) and C-reactive protein (r = 0.174, *P* < 0.001).

### DNAmAAs and mortality in participants with diabetes and pre-diabetes

In the fully adjusted Cox proportional hazards models, significant associations were found between multiple DNAmAAs and all-cause mortality (Table 2). Notably, for every 5-unit increase in AgeAccelGrim2 in the total population, it was associated with 35% increased risk of all-cause death (95% CI: 1.23–1.49), 50% increased risk of cardiovascular death (95% CI: 1.25–1.80) and 30% increased risk of non-cardiovascular death (95% CI: 1.16–1.46). When analyzed as a categorical variable, the highest AgeAccelGrim2 tertile was associated with a 62% increase in the risk of all-cause mortality (95% CI: 1.30–2.02), a 80% increase in the risk of cardiovascular mortality (95% CI: 1.19–2.72), and a 55% increase in the risk of non-cardiovascular mortality (95% CI: 1.19–2.01) compared to the lowest AgeAccelGrim2 tertile (Table 2). These associations were also significant in participants with diabetes and pre-diabetes (Table 2 & Table 3). Additionally, the results from sensitivity analyses were broadly consistent with those of the initial analyses, supporting the robustness of our findings (eTable 6—eTable 10). AgeAccelGrim2 was also significantly associated with mortality in non-diabetes populations, with HRs demonstrating predictive power similar to that observed in diabetes populations (eTable 11).

**Table 2:** Associations between DNAmAA and mortality outcomes among participants with diabetes and pre-diabetes

	Overall participants		Participants with diabetes		Participants with pre-diabetes	
	HR (95% CI)	*P*	HR (95% CI)	*P*	HR (95% CI)	*P*
Overall survival	662 deaths		347 deaths		315 deaths	
HorvathAge Accel	1.072 (0.998, 1.151)	0.056	1.035 (0.939, 1.142)	0.485	1.094 (0.985, 1.215)	0.092
HannumAge Accel	**1.081 (1.003, 1.165)**	**0.042**	1.046 (0.947, 1.156)	0.375	1.090 (0.973, 1.220)	0.140
SkinBloodAge Accel	0.995 (0.920, 1.075)	0.898	0.972 (0.881, 1.073)	0.580	0.990 (0.876, 1.119)	0.872
PhenoAge Accel	**1.106 (1.043, 1.173)**	**0.001**	1.039 (0.962, 1.122)	0.332	**1.184 (1.080, 1.299)**	** < 0.001**
AgeAccelGrim2	**1.354 (1.230, 1.491)**	** < 0.001**	**1.309 (1.142, 1.500)**	** < 0.001**	**1.345 (1.171, 1.545)**	** < 0.001**
ZhangAge Accel	1.102 (0.879, 1.381)	0.401	0.996 (0.753, 1.316)	0.976	1.170 (0.813, 1.685)	0.398
LinAge Accel	1.018 (0.968, 1.071)	0.494	0.988 (0.923, 1.057)	0.722	1.038 (0.960, 1.122)	0.347
WeidnerAge Accel	1.004 (0.960, 1.051)	0.847	1.002 (0.937, 1.071)	0.954	1.020 (0.958, 1.087)	0.528
Vidal-BraloAge Accel	**1.098 (1.016, 1.186)**	**0.018**	1.083 (0.973, 1.207)	0.145	1.114 (0.994, 1.250)	0.064
DunedinPoAm	**1.157 (1.055, 1.270)**	**0.002**	1.096 (0.967, 1.243)	0.150	**1.184 (1.032, 1.358)**	**0.016**
Cardiovascular death	187 deaths		103 deaths		84 deaths	
HorvathAge Accel	0.990 (0.868, 1.129)	0.880	1.068 (0.893, 1.278)	0.470	0.827 (0.672, 1.016)	0.071
HannumAge Accel	1.052 (0.914, 1.211)	0.478	1.032 (0.863, 1.235)	0.728	1.015 (0.812, 1.270)	0.893
SkinBloodAge Accel	0.998 (0.863, 1.153)	0.973	1.007 (0.835, 1.215)	0.941	0.898 (0.718, 1.124)	0.347
PhenoAge Accel	1.105 (0.987, 1.237)	0.082	1.081 (0.938, 1.246)	0.284	1.097 (0.910, 1.322)	0.330
AgeAccelGrim2	**1.501 (1.252, 1.799)**	** < 0.001**	**1.411 (1.100, 1.810)**	**0.007**	**1.524 (1.169, 1.985)**	**0.002**
ZhangAge Accel	0.984 (0.663, 1.458)	0.935	0.981 (0.601, 1.600)	0.937	0.794 (0.427, 1.475)	0.466
LinAge Accel	0.991 (0.901, 1.090)	0.853	1.006 (0.889, 1.137)	0.929	0.910 (0.779, 1.064)	0.239
WeidnerAge Accel	1.014 (0.933, 1.102)	0.742	1.036 (0.915, 1.172)	0.578	1.003 (0.887, 1.133)	0.966
Vidal-BraloAge Accel	1.005 (0.870, 1.160)	0.948	1.041 (0.858, 1.264)	0.682	0.911 (0.723, 1.147)	0.429
DunedinPoAm	1.235 (1.041, 1.465)	0.160	1.062 (0.842, 1.340)	0.609	**1.357 (1.054, 1.747)**	**0.018**
Non-cardiovascular death	475 deaths		244 deaths		231 deaths	
HorvathAge Accel	**1.103 (1.015, 1.200)**	**0.022**	1.020 (0.907, 1.147)	0.741	**1.200 (1.065, 1.352)**	**0.003**
HannumAge Accel	1.091 (0.998, 1.192)	0.054	1.047 (0.929, 1.182)	0.451	1.118 (0.980, 1.275)	0.096
SkinBloodAge Accel	0.991 (0.904, 1.087)	0.855	0.955 (0.851, 1.072)	0.437	1.022 (0.884, 1.182)	0.765
PhenoAge Accel	**1.103 (1.029, 1.182)**	**0.006**	1.018 (0.928, 1.117)	0.702	**1.207 (1.085, 1.342)**	**0.001**
AgeAccelGrim2	**1.299 (1.160, 1.456)**	** < 0.001**	**1.263 (1.073, 1.486)**	**0.005**	**1.288 (1.095, 1.515)**	**0.002**
ZhangAge Accel	1.147 (0.874, 1.506)	0.323	0.992 (0.708, 1.390)	0.964	1.358 (0.874, 2.110)	0.173
LinAge Accel	1.027 (0.968, 1.090)	0.372	0.979 (0.902, 1.063)	0.618	1.082 (0.990, 1.184)	0.083
WeidnerAge Accel	0.999 (0.946, 1.054)	0.957	0.987 (0.911, 1.069)	0.747	1.024 (0.951, 1.103)	0.524
Vidal-BraloAge Accel	**1.134 (1.035, 1.243)**	**0.007**	1.100 (0.966, 1.252)	0.149	**1.195 (1.047, 1.364)**	**0.008**
DunedinPoAm	**1.130 (1.012, 1.261)**	**0.030**	1.112 (0.958, 1.290)	0.163	1.120 (0.952, 1.318)	0.173

Cox proportional hazards models were adjusted for chronological age, sex, ethnicity, poverty income ratio, smoking status, body mass index, geriatric nutritional risk index, cardiovascular disease, hypertension, hyperlipidemia, and chronic kidney disease

Bold indicates significant at *P* < 0.05

CI, confidence interval; DNAmAA, DNA-methylation age acceleration; HR, hazard ratio

**Table 3:** Associations between AgeAccelGrim2 tertiles and mortality outcomes among participants with diabetes and pre-diabetes

	Overall participants		Participants with diabetes		Participants with pre-diabetes	
	HR (95% CI)	*P*	HR (95% CI)	*P*	HR (95% CI)	*P*
Overall survival	662 deaths		347 deaths		315 deaths	
AgeAccelGrim2 T1	Reference	Reference	Reference	Reference	Reference	Reference
AgeAccelGrim2 T2	1.113 (0.906, 1.367)	0.310	1.022 (0.775, 1.349)	0.875	1.171 (0.863, 1.590)	0.310
AgeAccelGrim2 T3	1.619 (1.298, 2.019)	< 0.001	1.515 (1.128, 2.036)	0.006	1.568 (1.119, 2.199)	0.009
Cardiovascular Death	187 deaths		103 deaths		84 deaths	
AgeAccelGrim2 T1	Reference	Reference	Reference	Reference	Reference	Reference
AgeAccelGrim2 T2	1.039 (0.700, 1.542)	0.849	1.001 (0.594, 1.687)	0.997	1.153 (0.616, 2.159)	0.791
AgeAccelGrim2 T3	1.802 (1.194, 2.720)	0.005	1.815 (1.065, 3.094)	0.028	1.995 (1.026, 3.878)	0.042
Non-Cardiovascular Death	475 deaths		244 deaths		231 deaths	
AgeAccelGrim2 T1	Reference	Reference	Reference	Reference	Reference	Reference
AgeAccelGrim2 T2	1.139 (0.895, 1.449)	0.290	1.029 (0.741, 1.428)	0.865	1.176 (0.829, 1.668)	0.365
AgeAccelGrim2 T2	1.548 (1.192, 2.012)	0.001	1.396 (0.978, 1.993)	0.066	1.450 (0.978, 2.149)	0.065

Cox proportional hazards models were adjusted for chronological age, sex, ethnicity, poverty income ratio, smoking status, body mass index, geriatric nutritional risk index, cardiovascular disease, hypertension, hyperlipidemia, and chronic kidney disease

CI, confidence interval; HR, hazard ratio

**Fig. 2:**
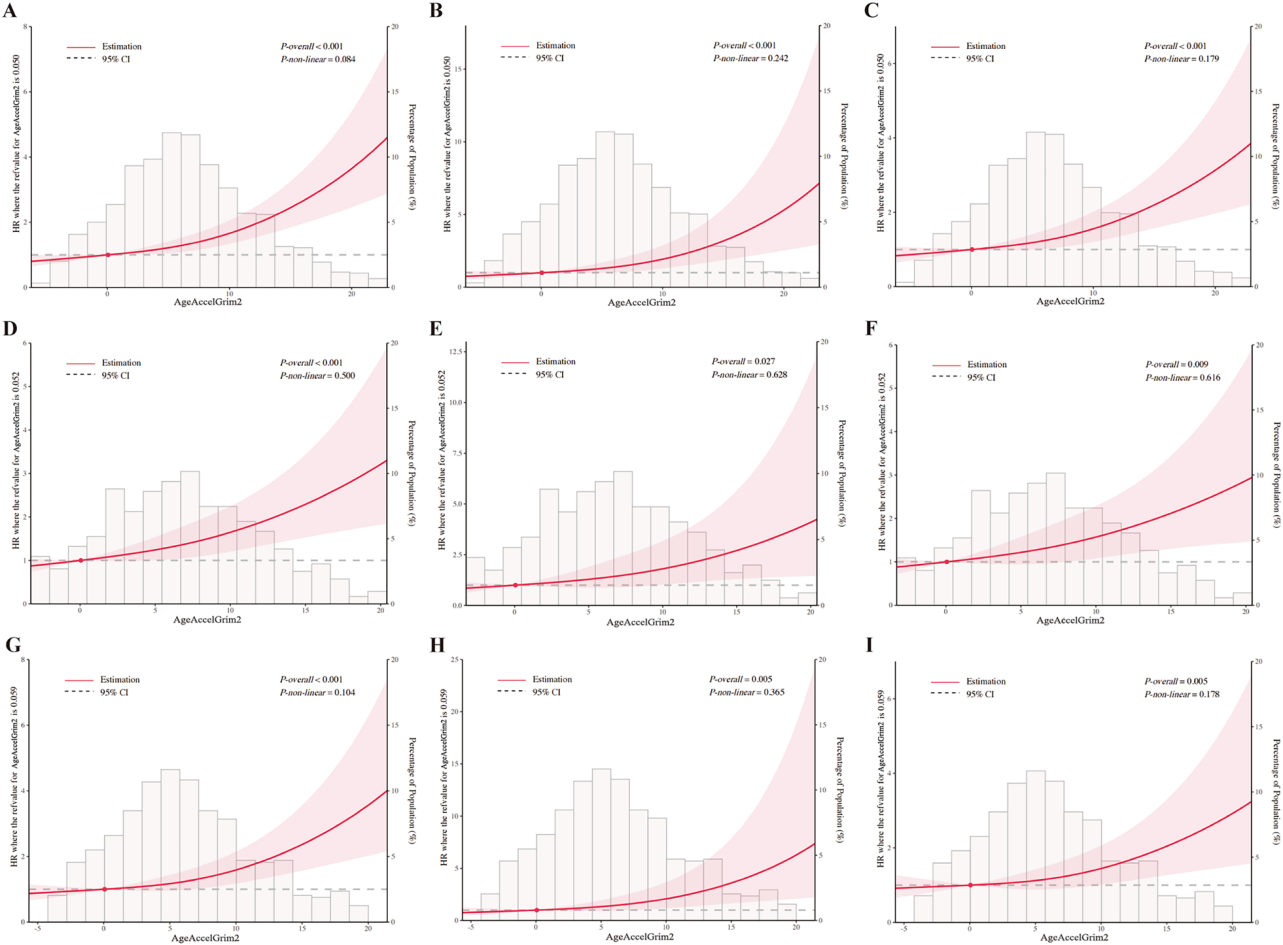
Restricted cubic spline analyses for associations between AgeAccelGrim2 and mortality outcomes. All-cause **A**, cardiovascular **B**, and non-cardiovascular **C** mortality for overall participants. All-cause **D**, cardiovascular **E**, and non-cardiovascular **F** mortality for diabetes participants. All-cause **G**, cardiovascular **H**, and non-cardiovascular **I** mortality for pre-diabetes participants. Abbreviations: CI, confidence interval; HR, hazard ratio

As shown in Fig. 2, the results of the RCS analysis further confirmed the relationships between AgeAccelGrim2 and all-cause, cardiovascular, and non-cardiovascular mortality outcomes (all *P*-overall < 0.05 and *P*-non-linear > 0.05). Although other DNAmAAs (including HannumAA, PhenoAA and Vidal-BraloAA) were also significantly associated with all-cause mortality, their relative hazard ratios and statistical significance were weaker than AgeAccelGrim2, and they showed inconsistent results in participants with diabetes and pre-diabetes. Moreover, the results of the subgroup analyses showed no significant interactions between AgeAccelGrim2 and mortality outcomes across the various subgroups in participants with diabetes (eTable 12) and pre-diabetes (eTable 13).

### Mediation of DNAmAA in mortality risk

**Fig. 3:**
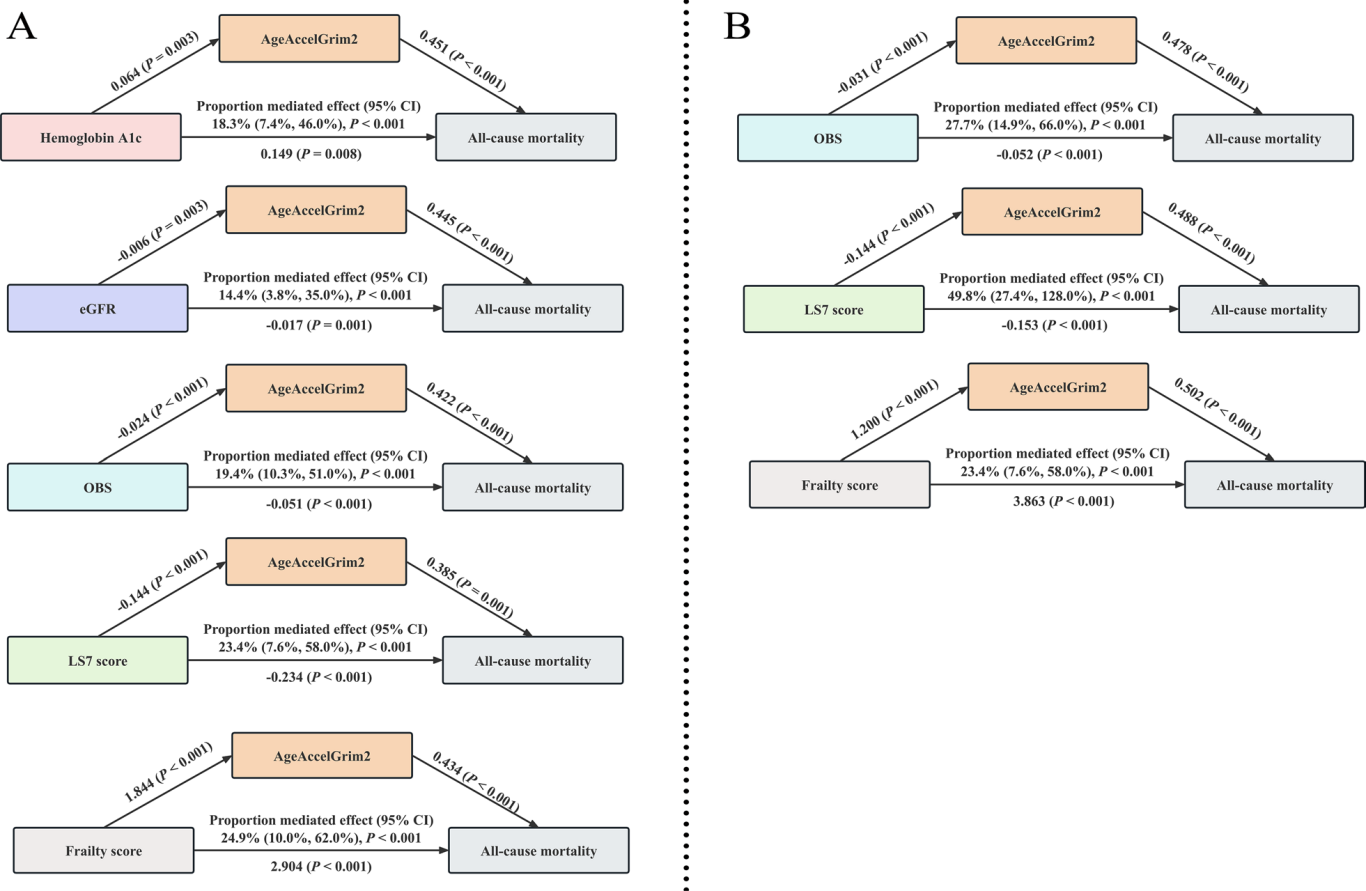
Mediation effects of AgeAccelGrim2 on the relationships between health-related exposures with all-cause mortality risk in participants with diabetes **A** and pre-diabetes **B**. Abbreviations: CI, confidence interval; eGFR, estimated glomerular filtration rate; LS7, Life’s Simple 7; OBS, Oxidative Balance Score

Our analysis identified AgeAccelGrim2 as a significant mediator in the relationships between health-related exposures with all-cause mortality in both diabetes and pre-diabetes populations (eTable 14). In the diabetes group (Fig. 3A), AgeAccelGrim2 showed its mediation effects through five pathways, with the strongest three corresponding to the following exposures: (1) frailty score, where AgeAccelGrim2 mediated 24.9% of the association (95% CI: 10.0%-62.0%); (2) LS7 score, where AgeAccelGrim2 mediated 23.4% (95% CI: 7.6%-58.0%) of the association between AgeAccelGrim2 and all-cause mortality; (3) OBS, where AgeAccelGrim2 mediated 19.4% of the association (95% CI: 10.3%-51.0%). AgeAccelGrim2 significantly mediated 18.3% and 14.4% of the associations between Hemoglobin A1c and eGFR and all-cause mortality, respectively. In the pre-diabetes group (Fig. 3B), AgeAccelGrim2 as a mediator variable mediates the associations between LS7 score, OBS, frailty score with all-cause mortality, respectively. (LS7 score: 49.8%, 95% CI: 27.4%-128.0%; OBS: 27.7%, 95% CI: 14.9%-66.0%; frailty score: 13.9%, 95% CI: 6.7%-46.0%).

## Discussion

### Main findings

This cohort study aimed to evaluate the associations of DNAmAAs with the risk of all-cause/cardiovascular/non-cardiovascular mortality in populations with diabetes and pre-diabetes and to explore potential mediating mechanisms. AgeAccelGrim2 showed stronger predictive performance than the other DNAmAAs, especially as each 5-unit increase in AgeAccelGrim2 was associated with a 35%, 50%, and 30% increase in the risk of all-cause, cardiovascular, and non-cardiovascular mortality, respectively. Additionally, although other DNAmAAs (e.g., HannumAA) also showed significant associations with the risk of all-cause mortality, their effect strengths were weaker and inconsistent in the diabetes and pre-diabetes populations. This suggests that AgeAccelGrim2 may be a more reliable biomarker for assessing the risk of mortality in this high-risk population. Moreover, our mediation analysis revealed that AgeAccelGrim2 statistically mediates the associations between several important health-related exposures (including LS7 score, frailty score, and OBS) and the risk of all-cause mortality in both diabetes and pre-diabetes populations. This suggests that AgeAccelGrim2 may serve as a biomarker reflecting the cumulative biological impact of these exposures on mortality risk. Whether AgeAccelGrim2 could be a viable target for interventions requires further investigation in future studies.

### Comparison with the existing literature

T2D is a complex metabolic disorder with multifaceted pathogenesis, in which DNAm plays a critical role in disease development and progression. Research has demonstrated that significant DNAm alterations occur in key metabolic tissues, including pancreatic islets, skeletal muscle, adipose tissue, and the liver of individuals with T2D. These epigenetic modifications regulate gene expression patterns, thereby disrupting insulin secretion and metabolic homeostasis. For instance, in the pancreatic islets of T2D patients, promoter regions of genes critical for *β*-cell function, such as INS, PDX1, PPARGC1A, and GLP1R, exhibit hypermethylation, leading to their transcriptional silencing and subsequent impairment of insulin secretion [6]. Although blood samples reflect systemic changes rather than tissue-specific alterations, there is growing evidence suggesting a direct relationship between white blood cell methylation and diabetes pathology. White blood cells are key players in chronic low-grade inflammation and immune dysregulation, which are hallmarks of diabetes. Methylation changes in immune-related genes (e.g., TXNIP, ABCG1) have been associated with inflammatory pathways, insulin signaling, and glucose metabolism, thereby contributing to diabetes progression [34].

Diabetes, a metabolic disease closely linked to aging. Fraszczyk et al. investigated DNA DNAm trajectories, epigenetic age, and age acceleration in relation to T2D onset using four epigenetic clocks (GrimAge, Hannum, Horvath, PhenoAge) in a nested case–control study, and revealed distinct DNAm patterns and accelerated epigenetic aging in T2D cases up to 10 years before diagnosis, highlighting the potential of DNAm markers and epigenetic clocks for early prediction of T2D and age-related diseases [18]. In young adults, Kim et al. found that both increased and accelerated GrimAge are associated with a higher risk of T2D and partially mediated the relationship between cumulative obesity and T2D development, highlighting its potential as a biomarker for T2D risk prediction [17]. In addition, Vetter et al. found that DNAmAA, particularly measured by the 7-CpG clock, is associated with an increased risk of diabetes complications in male patients with T2D [19]. Consistent with our findings, Sabbatinelli et al. reported that deceased individuals with diabetes exhibited significantly higher DNAmPhenoAge and accelerated DunedinPoAm pace of aging [21]. While their study did not directly examine DNAmAA, their use of age-adjusted models for DNAmAge allowed for a comparable assessment of mortality risk. This approach aligns with the findings of Krieger et al., [35] who demonstrated that DNAmAge and DNAmAA can provide overlapping insights into mortality risk when age is appropriately controlled for in statistical models.

Although Sabbatinelli et al. identified DNAmPhenoAge and accelerated DunedinPoAm as independent predictors of increased mortality risk in T2D patients, their study was limited by its small-sample case–control design, and the predictive utility of these DNAmAA measures has not yet been thoroughly validated in diabetes- and pre-diabetes-specific populations. In contrast, our study, leveraging a larger cohort, further substantiates the predictive efficacy of DNAmAA, particularly AgeAccelGrim2, in assessing mortality risk in individuals with diabetes. Notably, this is the first study to demonstrate that DNAmAA may also serve as a potential biomarker for predicting mortality risk in pre-diabetes populations. These findings not only reinforce the utility of DNAmAA in mortality risk prediction but also broaden its potential applications, offering valuable insights for future research in this field.

### Mediation mechanisms

This study revealed that AgeAccelGrim2 significantly mediated the associations between OBS, LS7 score, and frailty score and all-cause mortality in both diabetic and pre-diabetic populations, with mediating effects ranging from 10 to 60%. However, it is important to note that these findings represent statistical mediation and do not imply a causal relationship. The potential biological mechanisms underlying these associations remain hypothetical. Possible pathways may include: (1) Oxidative stress, as a key factor in cellular damage, promotes cellular dysfunction and accelerated aging by inducing DNA damage, protein and lipid oxidation [36, 37]. (2) Frailty may lead to chronic inflammation and immune senescence, which further exacerbate the decline in physiological functions [38, 39]. (3) Unhealthy lifestyles (e.g., smoking, low physical activity) may worsen metabolic control and increase oxidative stress and inflammatory responses [40, 41]. These pathways may explain the observed statistical associations, but causal inference cannot be drawn from this observational mediation analysis.

### Clinical implications and future directions

Our study proves the potential of AgeAccelGrim2 as a robust biomarker for assessing risk of all-cause/cardiovascular/non-cardiovascular mortality in individuals with diabetes and pre-diabetes. AgeAccelGrim2 could be integrated into current models to improve performance of predicting mortality in diabetes/pre-diabetes populations. However, the observed mediating effects of AgeAccelGrim2 with health-related exposures (such as LS7 score, frailty score, and OBS) should not be interpreted as evidence of a causal pathway. As such, any potential interventions targeting biological aging or DNAmAA remain hypothetical and require further investigation. For diabetes/pre-diabetes patients, reducing oxidative stress, improving lifestyle, strengthening strength training, and emphasizing nutritional supplementation may have potential to influence biological aging or mortality risk. However, our study does not directly evaluate the impact of these interventions on epigenetic clocks or mortality outcomes. Further research, including interventional trials, is warranted to determine whether such strategies can effectively slow epigenetic age acceleration and reduce mortality risk in populations with diabetes or pre-diabetes.

Future studies are critical to validate the role of AgeAccelGrim2 as a clinical biomarker in diabetes/pre-diabetes populations. Although our results are encouraging, the generality of AgeAccelGrim2 still requires further confirmation in different populations (including different ethnic groups, different age groups, different settings, and different regions) through larger and more diverse cohort studies. Furthermore, prospective studies are an exciting direction for future research to observe how lifestyle improvements or other interventions affect the biological aging of diabetes/pre-diabetes individuals and whether they can effectively reduce mortality.

### Limitations

Several limitations were identified in this study. First, the NHANES data are exclusively based on the USA, which may limit the generalizability of the findings to populations in other regions. Second, although multiple confounders were considered in this analysis, the retrospective observational study design means that some important variables (e.g., environmental exposures, genetic factors) may have been overlooked. Third, while our results suggested that AgeAccelGrim2 mediated the relationship between exposures and mortality outcomes, these exposures were only measured at baseline. Consequently, the dynamic changes in these exposures over time were not taken into account, which may further impact our results. Fourth, although this study provides reliable evidence for DNAmAA as a biomarker in populations with diabetes and pre-diabetes, external validation in independent cohorts would enhance the robustness of the findings. Fifth, we utilized DunedinPoAm instead of the more reliable version of DunedinPACE due to data availability constraints in NHANES, which may somewhat limit the usability of our findings. In addition, some of our results showed wide CIs, which might be attributed to the limited sample size and subgroup stratification. Future studies with larger sample sizes are needed to address this limitation. Sixth, multiple statistical tests were performed in this study, and no formal corrections for multiple comparisons (e.g., Bonferroni adjustment) were applied due to the exploratory nature of the analyses. Therefore, the possibility of type I error cannot be excluded. To enhance transparency, all p-values are reported with full precision in Supplementary Excel file, enabling readers to perform their own multiple testing corrections if needed. Future studies with larger sample sizes are warranted to validate these findings, incorporating appropriate multiple testing corrections to ensure robust results. Last, we conducted sensitivity analyses by excluding participants who died within two years of follow-up; however, the possibility of reverse causality cannot be entirely ruled out. Further rigorous prospective studies are needed to address these limitations.

## Conclusion

DNAmAA can serve as a potential biomarker for risk of all-cause/cardiovascular/non-cardiovascular mortality in both diabetes and pre-diabetes populations, with AgeAccelGrim2 standing out as particularly significant. While AgeAccelGrim2 demonstrated statistical mediation effects in the relationship between health-related exposures (OBS, LS7 score, frailty score) and all-cause mortality, these findings do not establish a causal pathway. These findings highlight the potential importance of biological aging in pre-diabetes/diabetes-related mortality and suggest that AgeAccelGrim2 may offer insights into future research directions. However, any intervention strategies targeting DNAmAA remain speculative and require validation in prospective interventional studies.

## Supplementary Information

Supplementary material 1

Supplementary material 2

## Data Availability

No datasets were generated or analyzed during the current study.

## Abbreviations

ASCVD: Atherosclerotic cardiovascular disease
BMI: Body mass index
CI: Confidence interval
CKD: Chronic kidney disease
DNAm: DNA-methylation
DNAmAA: DNA-methylation age acceleration
DNAmAge: DNA-methylation age
eGFR: Estimated glomerular filtration rate
GNRI: Geriatric nutritional risk index
HR: Hazard ratio
LS7: Life’s Simple 7
NHANES: National Health and Nutrition Examination Survey
OBS: Oxidative balance score
PIR: Poverty-to-income ratio
RCS: Restricted cubic spline
SD: Standard deviation
SII: Systemic immune inflammation index
UACR: Urine albumin-to-creatinine ratio
