# Supplementary material for: Unveiling the impact of DNA-methylation age acceleration on mortality risk in diabetes and pre-diabetes: insights from the US NHANES program
Source: Clin Epigenetics. 2025 May 16;17:81. doi: 10.1186/s13148-025-01886-0 (PMC12083022; doi:10.1186/s13148-025-01886-0)
Supplement: Supplementary file 2 — Supplementary material 2 [file 13148_2025_1886_MOESM2_ESM.pdf]

SUPPLEMENTARY FILES

Content

eMethod……………………………………………………………..……..……….………Page 2-13

eTable 1. Strengthening the Reporting of Observational Studies in Epidemiology Checklist for this cohort study…………………………………………….………………..……...Page 14-15

eTable 2. Percentage of missing value……………..……………………....…………....Page 16

eTable 3. Baseline characteristics of participants with diabetes……………………...…....Page 17

eTable 4. Baseline characteristics of participants with pre-diabetes…………………....…Page 18

eTable 5. DNAmAge and DNAmAA in participants with diabetes and pre-diabetes.....….Page 19

eTable 6. Sensitivity analysis adjusting for all covariates listed in Table 1..........…....Page 20-21

eTable 7. Sensitivity analysis after excluding participants with missing variables….....Page 22-23

eTable 8. Sensitivity analysis after excluding participants with missing variables for associations between GrimAA tertiles and mortality outcomes..Page 24

eTable 9. Sensitivity analysis after excluding participants who died within 2-year follow-up…………………………………………………………………..……..Page 25-26

eTable 10. Sensitivity analysis after excluding participants who died within 2-year follow-up for associations between GrimAA tertiles and mortality outcomes...Page 27

eTable 11. Associations between DNAmAA and mortality outcomes among general participants without diabetes/prediabetes..Page 28

eTable 12. Subgroup analysis for associations between Grim age acceleration and mortality outcomes in diabetes participants…………………………………………..…………Page 29

eTable 13. Subgroup analysis for associations between Grim age acceleration and mortality outcomes in pre-diabetes participants…………………………………..……….....…Page 30

eTable 14. Mediated effects of all mediators on the association Grim age acceleration and all-cause mortality among participants with diabetes and pre-diabetes……………….....…Page 31-32

eFigure 1. Flowchart of this study…………………………….…….………....…...Page 33

eFigure 2. Comparison the predictive performance of AgeAccelGrim2 and DunedinPoAm for mortality outcomes using ROC curve analysis...,Page 34

eFigure 3. The correlation analyses between each DNA-methylation age in populations with diabetes (A) and pre-diabetes (B)…………………………………………...………..Page 35

eFigure 4. Partial correlation analyses between different DNA-methylation age accelerations and multiple variables in populations with diabetes (A) and pre-diabetes (B)………………..…..Page 36

eMethod

Blood pressure, weight and height were measured according to standard protocols in the mobile examination center (MEC). Blood and urine specimens were also collected in the MEC following the standard operating procedures, and specimens were processed, stored and shipped to University of Minnesota, Minneapolis, MN for analysis.

BMI was calculated as weight in kilograms divided by height in meters squared. Smoking status was classified into never smoker (smoked < 100 cigarettes in life), former smoker (smoked ≥ 100 cigarettes in life and smoke not at all now), or current smoker (smoked ≥ 100 cigarettes in life and smoke some days or every day). ASCVD was defined as a composite of self-reported doctor diagnosis of coronary heart disease, congestive heart failure, heart attack, and stroke. Hypertension was defined as self-reported doctor diagnosis of hypertension, an average systolic blood pressure ≥ 140 mmHg or/and an average diastolic blood pressure ≥ 90 mmHg, or use of antihypertensive drugs. Hyperlipidemia was defined as total cholesterol ≥ 200 mg/dL, high-density lipoprotein cholesterol ≤ 40 mg/dL in males and ≤ 50 mg/dL in females, or use of cholesterol-lowering drugs. CKD was defined as estimated glomerular filtration rate < 60 ml/min/1.73m^2 as calculated using the Chronic Kidney Disease Epidemiology Collaboration Equation.[1] UACR was calculated using the formula: UACR (mg/g) = urine albumin (ug/mL) / urine creatinine (mg/dL) × 100.

The OBS was derived by summing scores assigned to 20 components, informed by their established connections to oxidative stress, which included 16 nutrients and 4 lifestyle factors. These components were categorized as prooxidants and antioxidants as shown in eMethod Table X1.[2] To represent dietary and dietary supplement intake, the average of the two 24-hour recalls was calculated, and each nutrient's intake was determined by adding contributions from both diet and dietary supplements. The LS7 metrics, as defined by the AHA, are outlined in eMethod Table X2.1 & eMethod Table X2.2.[3] Each component of the LS7 metrics was assigned a score of 2 points for ideal health, 1 point for intermediate health, and 0 point for poor health. The total LS7 score ranged from 0 to 14 points, with higher scores indicating better cardiovascular health. The assessment of frailty was based on the frailty index score constructed by Searle and colleagues following a standard procedure.[4] The chosen variables for the frailty index were deficits associated with health status, and a total of 40 variables covering multiple systems were utilized to construct the frailty index, which are outlined in eMethod Table X3. Each deficit was assigned a value between 0 and 1 based on its severity, and the frailty index score was calculated as a ratio of deficits present out of the total number of possible deficits, generating a continuous score from total fitness (0) to total frailty (1). The GNRI was established by Bouillanne et al. to evaluate the risk of malnutrition in elderly patients, and the standard weight in this formula with the ideal weight was determined by the Lorentz formula (WLo).[5] Ideal weight was calculated from the WLo as follows: For men: Ideal weight = Height - 100 - [(Height - 150)/4]. For women: Ideal weight = Height - 100 - [(H - 150)/2.5]. The GNRI formula is as follows: GNRI = [1.489 × albumin (g/L)] + [41.7 × (weight/WLo)].

Procedures of DNAmAge models

HorvathAge: HorvathAge is defined using a penalized regression model to predict DNA methylation age (DNAm age) using the R package 'glmnet'. The analysis utilized 21,369 CpG probes present on both Illumina 450K and 27K platforms, with fewer than 10 missing values. The training datasets included various healthy tissue samples, primarily from TCGA, and other publicly available datasets. An elastic net regression approach was employed with an alpha parameter set to 0.5. The lambda value (0.0226) was determined through cross-validation. DNAm age is calculated as the predicted age from the regression model, correlating closely with chronological age. The model's accuracy was validated across multiple datasets, demonstrating a high correlation between DNAm age and chronological age.

HannumAge: Blood samples were collected from participants, and genomic DNA was extracted using the QIAGEN FlexiGene DNA Kit. DNA methylation levels for autosomal chromosomes were assessed using the Illumina Infinium HumanMethylation450 BeadChip. Nested linear models and F-tests were used to analyze the relationship between methylation levels and various covariates (e.g., gender, BMI, diabetes status). Methylation markers were annotated and tested for enrichment in specific genomic regions. A multivariate linear model was constructed using the Elastic Net algorithm to predict age based on methylation markers. Optimal regularization parameters were determined through cross-validation, and bootstrap analysis was conducted to refine the model. Genetic variants were tested for associations with aging-related methylation markers using nested linear models, incorporating relevant covariates. Deviance was computed by fitting linear models to remove trends due to covariates and identifying non-normal markers. The remaining markers' deviance was calculated based on their adjusted methylation values. Shannon entropy statistics were computed to assess the variability of the methylation data, adjusted for covariates. Genomic positions for CpG islands were obtained, and marker values were mapped to these regions to analyze methylation patterns.

SkinBloodAge: DNA methylation levels at specific CpG sites are measured using Illumina BeadChips, generating beta values. Then, an age transformation function is applied to these values, using logarithmic transformation for ages up to 20 and linear transformation for older ages. Next, an elastic net regression model is employed to relate the transformed age to the beta values, with parameters optimized through cross-validation. The predicted DNA methylation age is calculated using the regression coefficients, and the final SkinBloodAge is obtained by reversing the transformation. This process integrates biological data from various studies to provide an estimate of biological age based on DNA methylation patterns.

PhenoAge: PhenoAge is calculated using NHANES training data through a Cox penalized regression model that examines the risk of aging-related mortality from various diseases. The model regresses mortality hazards against forty-two clinical markers and chronological age to identify key variables for the phenotypic age score. Using ten-fold cross-validation, a penalization parameter (lambda) is selected to ensure a simpler model with fewer biomarkers, resulting in a lambda of 0.0192. This process identifies ten important variables, including chronological age, albumin, creatinine, glucose, C-reactive protein, lymphocyte percent, mean cell volume, red cell distribution width, alkaline phosphatase, and white blood cell count. These variables are then incorporated into a parametric proportional hazards model based on the Gompertz distribution to estimate the 10-year mortality risk for individuals. This mortality score

is converted into years. Finally, the phenotypic age estimate is regressed against DNA methylation data using elastic net regression, leading to the selection of 513 specific CpG sites to refine the age estimate.

DNAmGrimAge2: DNAmGrimAge2 was derived using a two-stage approach within the Framingham Heart Study (FHS) cohort, which is a large-scale longitudinal study that began in 1948 and initially focused on cardiovascular disease risk factors. In the first stage, DNAmlogCRP and DNAmlogA1C were developed as DNAm-based surrogate markers for the log scale of C-reactive protein and hemoglobin, respectively. These were built using 2,544 individuals from 939 pedigrees, with 2/3 used for training and 1/3 for testing. In the second stage, DNAmGrimAge2 was defined by adding chronological age, gender, DNAmlogCRP, DNAmlogA1C, and 8 other previously defined DNAm biomarkers including DNAm adrenomedulin, DNAm beta-2-microglobulin, DNAm cystatin-C, DNAm growth differentiation factor 15, DNAm leptin, DNAm plasminogen activator inhibitor 1, DNAm tissue inhibitor metalloproteinases 1, DNAm pack-years. The final model was calibrated so that the mean and variance of DNAm GrimAge2 match those of the age variable in the FHS training data, resulting in an estimate that can be intuitively interpreted in years.

ZhangAge: Fourteen DNA methylation data cohorts, comprising a total of 13,661 samples (13,402 from blood, 259 from saliva) covering an age range of 2 to 104 years, were analyzed. The data was generated using the Illumina HumanMethylation450 and EPIC (850K) arrays. After quality control, a set of 319,607 probes (the "No Pruned set") was obtained. A "Pruned probe set" with 128,405 probes was also generated to compare its performance in age prediction. The Generation Scotland (GS) and Systems Genomics of Parkinson's Disease (SGPD) cohorts were used to estimate the proportion of variance in chronological age explained by DNA methylation, using the restricted maximum likelihood (REML) method. Two methods, Elastic Net and BLUP, were used to build age prediction models based on the training sets. The performance of the models was evaluated using correlation and root mean square error (RMSE) in the test sets. Eight large DNA methylation cohorts were used to evaluate the impact of data transformation (power, M-value, arcsine square root, log) on age prediction accuracy, using only the BLUP method. The association between age acceleration residual (AAR) and mortality using Cox proportional hazards regression, with sensitivity analyses including cell count covariates, were examined.

LinAge: The calculation of LinAge involves data from the Lothian Birth Cohorts (LBC) of 1921 and 1936, which are follow-up studies aimed at understanding cognitive aging. Participants were assessed around the ages of 79 and 70, respectively, with a focus on initial follow-up waves to ensure comparability and avoid biases from repeated measures. Raw DNA methylation data from the LBC cohorts were processed to generate β-values, which reflect methylation levels. Samples with low detection rates were excluded, resulting in high-quality datasets for analysis. The epigenetic age predictors were developed from a set of 102 CpGs showing significant age-related changes. Two models were created: a 99-CpG model and a simpler 3-CpG model, which focused on specific CpG sites retrained using cross-validation on external datasets. The models were validated against several publicly available DNA methylation datasets to ensure robustness.

WeinderAge: WeidnerAge is calculated using a method that involves blood samples and DNAm profiling. Blood samples were collected from the HNR study and two departments of the University Hospital Aachen, with consent and ethical approval. DNAm profiles from blood samples generated with the HumanMethylation27 BeadChip platform were considered. Beta values were combined, and 102 age-related CpG (AR-CpG) sites were selected based on high Pearson correlation coefficients with age. A multivariate linear model was trained for these AR-CpGs, and leave-one-out cross-validation was used to estimate the model's performance. Nucleotide sequences around each AR-CpG were analyzed for transcription factor binding motifs and histone modifications. Gene Ontology classification was also performed. From the 102 AR-CpGs, a smaller set was selected based on high interquartile range variations in beta values. Recursive feature elimination was applied to select the most relevant CpGs for age prediction. Genomic DNA was isolated, bisulfite-converted, and pyrosequencing was performed to analyze specific CpG sites. A multivariate model was generated based on these results for age prediction. Beta values at the following three CpGs were used for age-prediction: (α) = cg02228185; (β) = cg25809905, and (γ) = a CpG site upstream of cg17861230 that revealed better correlation with age. The model was validated with an independent set of samples. The model is as follows: Predicted age (in years) = $38.0 - 26.4\alpha - 23.7\beta + 164.7\gamma$

Vidal-BraloAge: The DNAmAge measure was developed using data from 390 healthy Caucasian individuals over 20 years old, as part of a study by Weidner et al. in 2014. The goal was to create an age estimation model from blood DNA that works well with MS-SNuPE technology. From a pool of 102 CpG sites with strong correlations to age, a selection process using forward stepwise linear regression identified 8 CpGs that were the most informative and feasible for MS-SNuPE assays. These 8 CpGs were included in the DmAM model with a significance threshold of $P < 0.05$.The accuracy of this 8 CpG DmAM was evaluated using the original training set and three separate validation sets. One validation set was specifically used to compare the new DmAM with three other existing models, as it had not been used in their calibration. The influence of gender and smoking on the DmAM was also examined through multiple regression analysis.

DunedinPoAm: DNAm data from the Lothian Birth Cohorts (LBC1921 and LBC1936) were processed by background correction and conversion to β-values using the R minfi package. Low detection rate probes were excluded, and manual inspection removed poor quality samples, yielding two refined datasets for aging prediction. The age prediction models, a 99-CpG and a 3-CpG model, were developed from 102 CpGs showing significant age-related changes. The 99-CpG model was based on Illumina HumanMethylation450 BeadChips, with coefficients detailed in Supplementary Table 1. The 3-CpG model, focused on cg02228185 (ASPA), cg25809905 (ITGA2B), and cg17861230 (PDE4C), was retrained on Hannum et al.'s dataset using leave-one-out cross-validation. For validation, the 99-CpG and 3-CpG models were tested against 12 public DNAm datasets from NCBI GEO, all generated with Illumina HumanMethylation450 BeadChip. This process ensured the models' accuracy in estimating epigenetic age.

eMethod Table X1 Oxidative balance score assignment scheme

OBS components	Property	Male			Female		
		0	1	2	0	1	2
Dietary OBS components							
Dietary fiber (g/d)[1]	A	<14.25	14.25–21.20	≥21.20	<12.25	12.25–18.00	≥18.00
β-Carotene (RE/d)	A	<710.00	710.00–2178.50	≥2178.50	<839.50	839.50–2371.00	≥2371.00
Vitamin B2 (mg/d) [1]	A	<2.06	2.06–3.41	≥3.41	<1.68	1.68–3.04	≥3.04
Niacin (mg/d) [1]	A	<24.82	24.82–40.32	≥40.32	<18.64	18.64–33.92	≥33.92
Vitamin B6 (mg/d)[1]	A	<1.67	1.67–2.39	≥2.39	<1.31	1.31–1.90	≥1.90
Total folate (mcg/d) [1]	A	<389.50	389.50–903.50	≥903.50	<322.00	322.00–938.50	≥938.5
Vitamin B12 (mcg/d) [1]	A	<4.62	4.62–15.14	≥15.14	<3.71	3.71–15.33	≥15.33
Vitamin C (mg/d) [1]	A	<39.25	39.25–109.935	≥109.935	<40.41	40.41–115.32	≥115.32
Vitamin E (ATE) (mg/d)	A	<6.20	6.20–9.49	≥9.49	<5.25	5.25–8.24	≥8.24
Calcium (mg/d) [1]	A	<771.50	771.50–1219.00	≥1219.00	<802.50	802.50–1362.00	≥1362.00
Magnesium (mg/d)[1]	A	<274.00	274.00–386.00	≥386.00	<234.00	234.00–325.50	≥325.50
Zinc (mg/d) [1]	A	<11.12	11.12–19.73	≥19.73	<8.58	8.58–17.78	≥17.78
Copper (mg/d)[1]	A	<1.18	1.18–1.85	≥1.85	<1.02	1.02–1.62	≥1.62
Selenium (mcg/d)[1]	A	<108.10	108.10–164.20	≥164.20	<81.90	81.90–124.90	≥124.90
Total fat (g/d)[1]	P	≥90.18	61.50–90.18	<61.50	≥71.90	47.26–71.90	<47.26
Iron (mg/d)[1]	P	≥19.70	12.66–19.70	<12.66	≥16.99	10.65–16.99	<10.65
Lifestyle OBS components							
Physical activity (MET-min/wk)	A	<150	150–300	≥300	<150	150–300	≥300
Alcohol (drinks/d)	P	≥2 drinks/d	< 2 drinks/d	<12 drinks/y	≥1 drinks/d	< 1 drinks/d	<12 drinks/y
Body mass index (kg/m^2)	P	≥30.10	26.00–30.10	<26.00	≥31.40	25.80–31.40	<25.80

OBS components	Property	Male			Female		
		0	1	2	0	1	2
Cotinine (ng/mL)	P	≥0.062	0.011–0.062	<0.011	≥0.036	0.011–0.036	<0.011

Abbreviations: A, antioxidant; ATE, alpha-tocopherol equivalent; MET, metabolic equivalent; OBS, oxidative balance score; P, prooxidant; RE, retinol equivalent; Total intake, dietary plus supplement intakes; inclusion of supplemental intake based on the availability of supplemental intake information.

eMethod Table X2.1 Definition of Life's Simple 7 metrics [a]

Metric	Level of Cardiovascular Health		
	Poor	Intermediate	Ideal
Blood pressure	BP ≥ 140/90 mmHg	SBP 120 to 139 mmHg or DBP 80 to 89 mmHg or treated to < 120/80 mmHg	< 120/80 mmHg and not on antihypertensive medication
Glycemic status [b]	HbA1c ≥ 6.5%	HbA1c 5.7% to 6.4% or treated with insulin or oral medications to HbA1c < 5.7%	HbA1c < 5.7% and not on glucose-lowering medication
Total cholesterol	≥ 240 mg/dL	200 to 239 mg/dL or treated to < 200 mg/dL	< 200 mg/dL and not on lipid-lowering medication
BMI	≥ 30 kg/m^2	25 to 29.9 kg/m^2	< 25 kg/m^2
Smoking [c]	Current smoker who had smoked ≥ 100 cigarettes in their lifetime and currently smoke some days or every day	Former smoker who had smoked ≥ 100 cigarettes in their lifetime but did not currently smoke	Never smoker or had smoked < 100 cigarettes in their lifetime
Diet	HEI < 50	HEI 50 to 80	HEI > 80
Physical activity	No activity	1 to 149 minutes moderate/vigorous per week	≥ 150 minutes moderate/vigorous per week

[a]The AHA definitions for poor, intermediate, and ideal health were used for blood pressure, cholesterol, BMI, and physical activity; modified definitions were used for glycemic status, smoking, and diet in NHANES.

[b]Glycemic status: AHA defined poor health as FPG ≥ 126 mg/dL or $HbA1_C$ ≥ 7%, intermediate health as FPG 100 to 125 mg/dL or $HbA1_C$ < 7%, and ideal health as FPG < 100 mg/dL.

[c]Smoking: AHA defined poor health as current smoker, intermediate health as quit smoking < 12 months, and ideal health as never smoker or quit smoking ≥ 12 months.

Abbreviations: AHA, American Heart Association; BP, blood pressure; BMI, body mass index; DBP, diastolic blood pressure; FPG, fasting plasma glucose; $HbA1_C$, hemoglobin A1c; HEI, Healthy Eating Index; NHANES, National Health and Nutrition Examination Survey; SBP, systolic blood pressure.

eMethod Table X2.2 The components and scoring criteria of HEI-2015

HEI-2015 Components	Range of Points	Minimum Scoring Standard	Maximum Scoring Standard
**Adequacy Components** (higher score indicates higher consumption)			
Total Fruits	0-5	0	0.8 cup equiv. /1000 kcal
Whole Fruits	0-5	0	0.4 cup equiv./1000 kcal
Total Vegetables	0-5	0	1.1 cup equiv. /1000 kcal
Greens and Beans	0-5	0	0.2 cup equiv. /1000 kcal
Total Protein Foods	0-5	0	2.5 oz equiv./1000 kcal
Seafood and Plant Proteins	0-5	0	0.8 oz equiv./1000 kcal
Dairy	0-10	0	1.3 cup equiv./1000 kcal
Whole Grains	0-10	0	1.5 oz equiv. /1000 kcal
Fatty Acids [b]	0-10	(PUFAs + MUFAs)/SFAs ≤1.2	(PUFAs + MUFAs)/SFAs ≥2.5
**Moderation Components** (higher score indicates lower consumption)			
Refined Grains	0-10	4.3 oz equiv./1000 kcal	1.8 oz equiv. /1000 kcal
Sodium	0-10	2.0 grams /1000 kcal	1.1 grams/1000 kcal
Added Sugars	0-10	26% of energy	6.5% of energy
Saturated Fats	0-10	16% of energy	8% of energy

[a]Intakes between the minimum and maximum standards are scored proportionately.

[b]Ratios of polyunsaturated and monounsaturated fatty acids (PUFAs and MUFAs) to saturated fatty acids (SFAs).

Abbreviations: HEI, Healthy Eating Index; MUFAs, monounsaturated fatty acids; PUFAs, polyunsaturated monounsaturated fatty acids; SFAs = saturated fatty acids.

eMethod Table X3 List of 49 variables included in the frailty index

Variable	Scoring	Proportion of missing value, n (%)
Cognition		
1. Experience confusion/memory problems	Yes = 1;<br>No = 0	10 (0.05%)
Dependence		
2. Managing money	No difficulty = 0;<br>Some difficulty = 0.33;<br>Much difficulty = 0.66;<br>Unable to do = 1	11,287 (52.23%)
3. Stooping, crouching, kneeling difficulty	No difficulty = 0;<br>Some difficulty = 0.33;<br>Much difficulty = 0.66;<br>Unable to do = 1	11,062 (51.19%)
4. Lifting or carrying difficulty	No difficulty = 0;<br>Some difficulty = 0.33;<br>Much difficulty = 0.66;<br>Unable to do = 1	11,079 (51.27%)
5. House chore difficulty	No difficulty = 0;<br>Some difficulty = 0.33;<br>Much difficulty = 0.66;<br>Unable to do = 1	11,209 (51.87%)
6. Preparing meals difficulty	No difficulty = 0;<br>Some difficulty = 0.33;<br>Much difficulty = 0.66;<br>Unable to do = 1	11,289 (52.24%)
7. Standing up from armless chair difficulty	No difficulty = 0;<br>Some difficulty = 0.33;<br>Much difficulty = 0.66;<br>Unable to do = 1	10,989 (50.85%)
8. Getting in and out of bed difficulty	No difficulty = 0;<br>Some difficulty = 0.33;<br>Much difficulty = 0.66;<br>Unable to do = 1	10,993 (50.87%)
9. Using fork, knife, drinking from cup difficulty	No difficulty = 0;<br>Some difficulty = 0.33;<br>Much difficulty = 0.66;<br>Unable to do = 1	10,986 (50.84%)
10. Dressing yourself difficulty	No difficulty = 0;<br>Some difficulty = 0.33;<br>Much difficulty = 0.66;<br>Unable to do = 1	10,990 (50.86%)
11. Standing for long periods difficulty	No difficulty = 0;<br>Some difficulty = 0.33;<br>Much difficulty = 0.66;	11,131 (51.51%)

	Unable to do = 1	
12. Grasp/holding small objects difficulty	No difficulty = 0;<br>Some difficulty = 0.33;<br>Much difficulty = 0.66;<br>Unable to do = 1	10,994 (50.88%)
13. Attending social event difficulty	No difficulty = 0;<br>Some difficulty = 0.33;<br>Much difficulty = 0.66;<br>Unable to do = 1	11,307 (52.33%)
14. Push or pull large objects difficulty	No difficulty = 0;<br>Some difficulty = 0.33;<br>Much difficulty = 0.66;<br>Unable to do = 1	13,082 (60.54%)
15. Walking for a quarter mile difficulty	No difficulty = 0;<br>Some difficulty = 0.33;<br>Much difficulty = 0.66;<br>Unable to do = 1	12,435 (57.55%)
16. Walking up 10 steps difficulty	No difficulty = 0;<br>Some difficulty = 0.33;<br>Much difficulty = 0.66;<br>Unable to do = 1	12,411 (57.43%)
17. Leisure activity at home difficulty	No difficulty = 0;<br>Some difficulty = 0.33;<br>Much difficulty = 0.66;<br>Unable to do = 1	11,001 (50.91%)
Depressive Symptoms		
18. Have little interest in doing things	1999-2004: Every day, nearly every day = 1, Most days = 0.75, about half the days = 0.50, less than half the days = 0.25, Not at all =0;<br>2005-2018: Nearly every day = 1, More than half the days = 0.66, Several days = 0.33, Not at all = 0	5,751 (26.61%)
19. Feeling down, depressed, or hopeless	1999-2004: Every day, nearly every day = 1, Most days = 0.75, about half the days = 0.50, less than half the days = 0.25, Not at all =0;<br>2005-2018: Nearly every day = 1, More than half the days = 0.66, Several days = 0.33, Not at all = 0	5,671 (26.24%)
20. Trouble sleeping or sleeping too much	1999-2004: Every night = 1, Nearly every night = 0.66, less often = 0.33, Not at all = 0<br>2005-2018: Nearly every day = 1, More than half the days = 0.66, Several days = 0.33, Not at all = 0	6,308 (29.19%)
21. Feeling tired or having little energy	Nearly every day = 1, More than half the days = 0.66, Several days = 0.33, Not at all = 0	6,413 (29.68%)
22. Poor appetite or overeating	1999-2004: Yes = 1, No = 0<br>2005:2018: Nearly every day = 1, More than half the days = 0.66, Several days = 0.33, Not at all = 0	6,305 (29.18%)
23. Feeling bad about yourself	1999-2004: Yes = 1, No = 0<br>2005:2018: Nearly every day = 1, More than half the days = 0.66, Several days = 0.33, Not at all = 0	6,312 (29.21%)
24.Trouble concentrating on things	1999-2004: Yes = 1, No = 0<br>2005:2018: Nearly every day = 1, More than half the days = 0.66, Several days = 0.33, Not at all = 0	6,309 (29.20%)

Comorbidities		
25. Arthritis	Yes = 1, No = 0	32 (0.15%)
26. Thyroid problems	Yes = 1, No = 0	33 (0.15%)
27. Chronic bronchitis	Yes = 1, No = 0	43 (0.20%)
28. Cancer	Yes = 1, No = 0	11 (0.05%)
29. Congestive heart failure	Yes = 1, No = 0	34 (0.16%)
30. Coronary heart disease	Yes = 1, No = 0	43 (0.20%)
31. Angina/Angina pectoris	Yes = 1, No = 0	58 (0.27%)
32. Heart attack	Yes = 1, No = 0	18 (0.08%)
33. Stroke	Yes = 1, No = 0	3 (0.01%)
34. Hypertension	Yes = 1, No = 0	58 (0.27%)
35. Diabetes	Yes = 1, No = 0	4 (0.02%)
36. Weak/failing kidneys	Yes = 1, No = 0	29 (0.13%)
37. Urinary Leakage	1999-2000: Yes = 1, No = 0<br>2001-2018: Greatly = 1, Very much = 0.75, Somewhat = 0.5, Only a little = 0.25	13,847 (64.08%)
Hospital Utilization and Access to Care		
38. Self-rated health	Fair, poor = 1, Excellent, Very good, good = 0	No missing value
39. Health now compared with 1 year ago	Worse = 1, About the same, better = 0	No missing value
40. Overnight hospital patient in past year	Yes = 1, No = 0	8 (0.04%)
41. Frequency of health care use during past year	None = 0, 1-4 = 0.5, 5 and more = 1	9 (0.04%)
42. Number of prescribed medications	None = 0, 1-4 = 0.5, 5 and more = 1	No missing value
Physical Anthropometry		
43. Body mass index	< 18.5, ≥30 = 1<br>25-30 = 0.5<br>18.5-25 = 0	No missing value
Laboratory Values		
44. Glycohemoglobin (%)	0%-5.7% = 0, >5.7% = 1	36 (0.17%)
45. Red blood cell count (million cells/mL)	MALE: 4.7-6.1 = 0, Other = 1 FEMALE: 4.2-5.4 = 0, Other = 1	41 (0.19%)
46. Hemoglobin (g/dL)	MALE: 13.5-18 = 0, Other = 1 FEMALE: 12-16 = 0, Other = 1	41 (0.19%)
47. Red cell distribution width (%)	11.6-14.6 = 0, Other = 1	41 (0.19%)
48. Lymphocyte percent (%)	20-40 = 0, Other = 1	80 (0.37%)
49. Segmented neutrophils percent (%)	40-80 = 0, Other = 1	80 (0.37%)

For participants with missing data on certain frailty-related items, the frailty score was calculated by dividing the total score of available frailty-related items by the total number of items for which the participant had data. This method was consistent with the procedure described by Hakeem et al. (Reference: Hakeem, F. F., Bernabé, E., & Sabbah, W. (2021). Association Between Oral Health and Frailty Among American Older Adults. Journal of the American Medical Directors Association, 22(3), 559–563.e2. https://doi.org/10.1016/j.jamda.2020.07.023).

eMethod References

1. Levey AS, Stevens LA, Schmid CH, Zhang YL, Castro AF 3rd, Feldman HI, Kusek JW, Eggers P, Van Lente F, Greene T, Coresh J; CKD-EPI (Chronic Kidney Disease Epidemiology Collaboration). A new equation to estimate glomerular filtration rate. *Ann Intern Med.* 2009;150(9):604-12. doi: 10.7326/0003-4819-150-9-200905050-00006.

2. Goodman M, Bostick RM, Dash C, Flanders WD, Mandel JS. Hypothesis: oxidative stress score as a combined measure of pro-oxidant and antioxidant exposures. *Ann Epidemiol.* 2007;17(5):394-9. doi: 10.1016/j.annepidem.2007.01.034.

3. Lloyd-Jones DM, Hong Y, Labarthe D, Mozaffarian D, Appel LJ, Van Horn L, Greenlund K, Daniels S, Nichol G, Tomaselli GF, Arnett DK, Fonarow GC, Ho PM, Lauer MS, Masoudi FA, Robertson RM, Roger V, Schwamm LH, Sorlie P, Yancy CW, Rosamond WD; American Heart Association Strategic Planning Task Force and Statistics Committee. Defining and setting national goals for cardiovascular health promotion and disease reduction: the American Heart Association's strategic Impact Goal through 2020 and beyond. *Circulation*. 2010;121(4):586-613. doi: 10.1161/CIRCULATIONAHA.109.192703.

4. Searle SD, Mitnitski A, Gahbauer EA, Gill TM, Rockwood K. A standard procedure for creating a frailty index. *BMC Geriatr.* 2008;8:24. doi: 10.1186/1471-2318-8-24.

5. Bouillanne O, Morineau G, Dupont C, Coulombel I, Vincent JP, Nicolis I, Benazeth S, Cynober L, Aussel C. Geriatric Nutritional Risk Index: a new index for evaluating at-risk elderly medical patients. *Am J Clin Nutr.* 2005;82(4):777-83. doi: 10.1093/ajcn/82.4.777.

eTable 1. Strengthening the Reporting of Observational Studies in Epidemiology Checklist for this cohort study

	Item No	Recommendation	Page No
**Title and abstract**	1	(*a*) Indicate the study's design with a commonly used term in the title or the abstract	1,3
		(*b*) Provide in the abstract an informative and balanced summary of what was done and what was found	3,4
**Introduction**			
Background/rationale	2	Explain the scientific background and rationale for the investigation being reported	5,6
Objectives	3	State specific objectives, including any prespecified hypotheses	7
**Methods**			
Study design	4	Present key elements of study design early in the paper	7,8,9
Setting	5	Describe the setting, locations, and relevant dates, including periods of recruitment, exposure, follow-up, and data collection	7,8
Participants	6	(*a*) Give the eligibility criteria, and the sources and methods of selection of participants. Describe methods of follow-up	7,8
		(*b*) For matched studies, give matching criteria and number of exposed and unexposed	N/A
Variables	7	Clearly define all outcomes, exposures, predictors, potential confounders, and effect modifiers. Give diagnostic criteria, if applicable	9
Data sources/ measurement	8*	For each variable of interest, give sources of data and details of methods of assessment (measurement). Describe comparability of assessment methods if there is more than one group	9
Bias	9	Describe any efforts to address potential sources of bias	9,10
Study size	10	Explain how the study size was arrived at	N/A
Quantitative variables	11	Explain how quantitative variables were handled in the analyses. If applicable, describe which groupings were chosen and why	10
Statistical methods	12	(*a*) Describe all statistical methods, including those used to control for confounding	10,11
		(*b*) Describe any methods used to examine subgroups and interactions	11
		(*c*) Explain how missing data were addressed	9,10
		(*d*) If applicable, explain how loss to follow-up was addressed	N/A
		(*e*) Describe any sensitivity analyses	11
**Results**			
Participants	13*	(a) Report numbers of individuals at each stage of study—eg numbers potentially eligible, examined for eligibility, confirmed eligible, included in the study, completing follow-up, and analysed	13
		(b) Give reasons for non-participation at each stage	13
		(c) Consider use of a flow diagram	13
Descriptive data	14*	(a) Give characteristics of study participants (eg demographic, clinical, social) and information on exposures and potential confounders	13
		(b) Indicate number of participants with missing data for each variable of interest	11
		(c) Summarise follow-up time (eg, average and total amount)	13

Outcome data	15*	Report numbers of outcome events or summary measures over time	13
Main results	16	(*a*) Give unadjusted estimates and, if applicable, confounder-adjusted estimates and their precision (eg, 95% confidence interval). Make clear which confounders were adjusted for and why they were included	13,14,15
		(*b*) Report category boundaries when continuous variables were categorized	13
		(*c*) If relevant, consider translating estimates of relative risk into absolute risk for a meaningful time period	13,14,15
Other analyses	17	Report other analyses done—eg analyses of subgroups and interactions, and sensitivity analyses	14,15
**Discussion**			
Key results	18	Summarise key results with reference to study objectives	18
Limitations	19	Discuss limitations of the study, taking into account sources of potential bias or imprecision. Discuss both direction and magnitude of any potential bias	24
Interpretation	20	Give a cautious overall interpretation of results considering objectives, limitations, multiplicity of analyses, results from similar studies, and other relevant evidence	21,22,23,24
Generalisability	21	Discuss the generalisability (external validity) of the study results	23,24
**Other information**			
Funding	22	Give the source of funding and the role of the funders for the present study and, if applicable, for the original study on which the present article is based	26

*Give information separately for exposed and unexposed groups

Note: An Explanation and Elaboration article discusses each checklist item and gives methodological background and published examples of transparent reporting. The STROBE checklist is best used in conjunction with this article (freely available on the Web sites of PLoS Medicine at http://www.plosmedicine.org/, Annals of Internal Medicine at http://www.annals.org/, and Epidemiology at http://www.epidem.com/). Information on the STROBE Initiative is available at http://www.strobe-statement.org

eTable 2. Percentage of missing value

Characteristics	Overall participants (n = 1199)		Survivors (n = 537)		Non-Survivors (n = 662)	
	N	Percentage	N	Percentage	N	Percentage
Poverty income ratio	139/1199	11.59	50/537	9.31	89/662	13.44
Body mass index	49/1199	4.09	8/537	1.49	41/662	6.19
Systolic blood pressure	56/1199	4.67	27/537	5.03	29/662	4.38
Diastolic blood pressure	79/1199	6.59	31/537	5.77	48/662	7.25
Hemoglobin A1c	1/1199	0.08	0/537	0	1/662	0.15
Total cholesterol	1/1199	0.08	1/537	0.19	0/662	0
HDL-C	3/1199	0.25	1/537	0.19	2/662	0.30
eGFR	3/1199	0.25	2/537	0.37	1/662	0.15
UACR	26/1199	2.17	4/537	0.74	22/662	3.32
Systemic immune inflammation index	8/1199	0.67	2/537	0.37	6/662	0.91
Geriatric Nutritional Risk Index	52/1199	4.34	10/537	1.86	42/662	6.34

Abbreviations: eGFR, estimated glomerular filtration rate; HDL-C, high-density lipoprotein cholesterol; UACR, urine albumin to creatinine ratio.

eTable 3. Baseline characteristics of participants with diabetes

Characteristics	All (n = 542)	Survivors (n = 195)	Non-survivors (n = 347)
Age, mean (SD), years	65.15 (0.86)	58.58 (0.91)	68.71 (0.91)
Male, n (%)	291 (53.69)	102 (48.97)	189 (48.66)
Ethnicity, n (%)			
Non-Hispanic White	135 (24.91)	30 (54.89)	105 (71.38)
Non-Hispanic Black	151 (27.86)	49 (16.86)	102 (14.64)
Mexican American	196 (36.16)	82 (7.58)	114 (4.63)
Hispanic and Other	60 (11.07)	34 (20.67)	26 (9.35)
Poverty income ratio, mean (SD)	2.51 (0.12)	2.82 (0.24)	2.34 (0.11)
Smoking status, n (%)			
Never smoker	264 (48.80)	96 (45.40)	168 (49.14)
Former smoker	203 (37.52)	74 (40.81)	129 (35.21)
Current smoker	74 (13.68)	24 (13.79)	50 (15.66)
Cardiovascular disease, n (%)	141 (26.01)	31 (21.09)	110 (36.92)
Hypertension, n (%)	408 (75.28)	131 (71.14)	277 (79.45)
Hypercholesterolemia, n (%)	465 (85.79)	165 (89.27)	300 (89.23)
Chronic kidney disease, n (%)	248 (46.53)	63 (29.92)	185 (49.00)
Body mass index, mean (SD), kg/m^2	31.56 (0.56)	31.40 (0.91)	31.65 (0.49)
Systolic blood pressure, mean (SD), mmHg	135.46 (1.22)	130.95 (2.51)	137.90 (1.55)
Diastolic blood pressure, mean (SD), mmHg	68.61 (1.03)	74.26 (1.15)	65.49 (1.58)
Hemoglobin A1c, mean (SD), %	7.50 (0.11)	7.24 (0.25)	7.64 (0.14)
Total cholesterol, mean (SD), mmol/L	5.32 (0.08)	5.29 (0.09)	5.33 (0.11)
HDL-C, mean (SD), mmol/L	1.17 (0.02)	1.18 (0.04)	1.16 (0.02)
eGFR, mean (SD), ml/min/1.73m^2	75.35 (1.79)	85.69 (1.82)	69.80 (2.10)
UACR, mean (SD), mg/g	185.80 (47.32)	87.14 (30.27)	239.66 (67.59)
SII, mean (SD)	582.13 (16.61)	561.85 (26.22)	593.28 (24.44)
C-reactive protein, mean (SD), mg/dl	0.71 (0.05)	0.63 (0.07)	0.76 (0.09)
Oxidative Balance Score, mean (SD)	16.80 (0.43)	18.58 (0.88)	15.84 (0.12)
Life's Simple 7 score, mean (SD)	5.30 (0.15)	5.76 (0.27)	5.06 (0.12)
Frailty score, mean (SD)	0.24 (0.01)	0.22 (0.01)	0.24 (0.01)
Geriatric Nutritional Risk Index, mean (SD)	122.84 (1.03)	122.81 (1.39)	122.59 (1.02)

Abbreviations: eGFR, estimated glomerular filtration rate; HDL-C, high-density lipoprotein cholesterol; UACR, urine albumin to creatinine ratio; SD, standard deviation; SII, Systemic immune inflammation index.

eTable 4. Baseline characteristics of participants with pre-diabetes

Characteristics	All (n = 657)	Survivors (n = 342)	Non-survivors (n = 315)
Age, mean (SD), years	63.62 (0.56)	58.79 (0.47)	70.33 (0.66)
Male, n (%)	330 (50.23)	164 (46.84)	166 (44.41)
Ethnicity, n (%)			
Non-Hispanic White	229 (34.86)	106 (71.73)	123 (75.70)
Non-Hispanic Black	162 (24.66)	78 (10.22)	84 (11.67)
Mexican American	196 (29.83)	112 (4.16)	84 (4.09)
Hispanic and Other	70 (10.65)	46 (13.89)	24 (8.54)
Poverty income ratio, mean (SD)	3.05 (0.11)	3.35 (0.17)	2.59 (0.14)
Smoking status, n (%)			
Never smoker	313 (47.64)	176 (43.38)	135 (40.93)
Former smoker	231 (35.16)	114 (36.26)	117 (38.82)
Current smoker	113 (17.2)	51 (20.36)	62 (20.25)
Cardiovascular disease, n (%)	107 (16.29)	31 (7.45)	76 (28.91)
Hypertension, n (%)	421 (64.08)	198 (56.56)	223 (69.02)
Hypercholesterolemia, n (%)	551 (83.87)	287 (87.01)	264 (88.08)
Chronic kidney disease, n (%)	164 (25.27)	47 (16.79)	117 (35.82)
Body mass index, mean (SD), kg/m^2	30.09 (0.32)	30.55 (0.50)	29.43 (0.42)
Systolic blood pressure, mean (SD), mmHg	135.04 (0.88)	132.57 (1.33)	138.42 (1.21)
Diastolic blood pressure, mean (SD), mmHg	73.28 (0.80)	76.49 (1.02)	68.77 (0.92)
Hemoglobin A1c, mean (SD), %	5.69 (0.02)	5.68 (0.02)	5.69 (0.02)
Total Cholesterol, mean (SD), mmol/L	5.69 (0.06)	5.71 (0.07)	5.67 (0.10)
HDL-Cholesterol, mean (SD), mmol/L	1.28 (0.02)	1.24 (0.03)	1.32 (0.03)
eGFR, mean (SD), ml/min/1.73m^2	79.48 (1.12)	83.89 (1.05)	73.36 (1.61)
UACR, mean (SD), mg/g	28.63 (4.17)	16.77 (2.15)	45.29 (9.15)
SII, mean (SD)	671.04 (35.82)	648.95 (51.10)	701.66 (45.69)
C-reactive protein, mean (SD), mg/dl	0.63 (0.09)	0.55 (0.08)	0.74 (0.17)
Oxidative Balance Score, mean (SD)	17.55 (0.41)	18.11 (0.67)	16.77 (0.17)
Life's Simple 7 score, mean (SD)	6.28 (0.13)	6.37 (0.14)	6.16 (0.17)
Frailty score, mean (SD)	0.16 (0.00)	0.14 (0.01)	0.17 (0.01)
Geriatric Nutritional Risk Index, mean (SD)	120.76 (0.58)	121.97 (0.92)	118.99 (0.77)

Abbreviations: eGFR, estimated glomerular filtration rate; HDL-C, high-density lipoprotein cholesterol; UACR, urine albumin to creatinine ratio; SD, standard deviation; SII, Systemic immune inflammation index.

eTable 5. DNAmAge and DNAmAA in participants with diabetes and pre-diabetes

	Overall participants (n = 1,199)			Participants with diabetes (n = 542)			Participants with pre-diabetes (n = 657)		
	All	Survivors	Non-Survivors	All	Survivors	Non-Survivors	All	Survivors	Non-Survivors
Chronological age, mean (SD), years	64.20 (0.46)	58.73 (0.41)	69.55 (0.53)	65.15 (0.86)	58.58 (0.91)	68.71 (0.91)	63.62 (0.56)	58.79 (0.47)	70.33 (0.66)
DNAmAge, mean (SD), years									
Horvath	65.62 (0.35)	61.51 (0.43)	69.65 (0.46)	66.24 (0.64)	60.99 (0.94)	69.08 (0.75)	65.25 (0.44)	61.70 (0.43)	70.18 (0.56)
Hannum	65.70 (0.38)	61.10 (0.46)	70.22 (0.41)	66.54 (0.64)	60.53 (0.89)	69.79 (0.67)	65.20 (0.53)	61.30 (0.49)	70.62 (0.60)
SkinBlood	62.92 (0.39)	58.42 (0.46)	67.34 (0.47)	63.95 (0.63)	58.04 (1.06)	67.15 (0.70)	62.31 (0.52)	58.56 (0.47)	67.52 (0.66)
Pheno	54.36 (0.44)	49.21 (0.49)	59.40 (0.52)	55.38 (0.69)	49.36 (0.96)	58.64 (0.74)	53.75 (0.54)	49.15 (0.53)	60.12 (0.63)
GrimV2	71.02 (0.36)	66.44 (0.33)	75.51 (0.43)	77.22 (0.67)	66.43 (0.71)	75.35 (0.64)	70.30 (0.39)	66.44 (0.34)	75.66 (0.47)
Zhang	66.23 (0.14)	64.37 (0.18)	68.05 (0.16)	66.54 (0.25)	64.12 (0.40)	67.84 (0.26)	66.04 (0.20)	64.46 (0.18)	68.23 (0.24)
Lin	55.59 (0.44)	50.55 (0.48)	60.52 (0.67)	56.22 (0.72)	50.53 (1.00)	59.30 (0.92)	55.20 (0.60)	50.56 (0.58)	61.64 (0.89)
Weidner	53.71 (0.53)	50.44 (0.58)	56.92 (0.74)	53.49 (0.77)	49.58 (0.71)	55.60 (1.14)	53.85 (0.65)	50.75 (0.80)	58.14 (1.00)
Vidal-Bralo	60.00 (0.31)	57.46 (0.43)	62.49 (0.34)	59.91 (0.50)	56.53 (0.54)	61.74 (0.56)	60.06 (0.43)	57.80 (0.56)	63.19 (0.43)
DNAmAA, mean (SD), years									
Horvath	0.24 (0.25)	0.10 (0.39)	0.37 (0.35)	-0.01 (0.41)	-0.73 (0.70)	0.38 (0.49)	0.43 (0.27)	0.54 (0.42)	0.29 (0.39)
Hannum	0.05 (0.27)	-0.22 (0.44)	0.30 (0.30)	-0.16 (0.37)	-1.02 (0.62)	0.30 (0.44)	0.23 (0.33)	0.21 (0.48)	0.27 (0.39)
SkinBlood	0.04 (0.25)	0.14 (0.37)	-0.06 (0.33)	0.01 (0.41)	-0.45 (0.61)	0.27 (0.49)	0.11 (0.32)	0.47 (0.42)	-0.40 (0.40)
Pheno	-0.03 (0.25)	-0.65 (0.41)	0.57 (0.39)	-0.27 (0.41)	-1.09 (0.68)	0.18 (0.54)	0.22 (0.32)	-0.23 (0.47)	0.85 (0.49)
GrimV2	-0.36 (0.25)	-1.19 (0.30)	0.45 (0.35)	-0.38 (0.31)	-1.66 (0.36)	0.32 (0.36)	-0.23 (0.33)	-0.79 (0.39)	0.54 (0.49)
Zhang	0.12 (0.08)	0.09 (0.15)	0.16 (0.11)	0.09 (0.14)	-0.16 (0.20)	0.22 (0.15)	0.15 (0.10)	0.20 (0.17)	0.09 (0.13)
Lin	0.03 (0.25)	0.12 (0.42)	-0.06 (0.41)	-0.50 (0.38)	-0.32 (0.82)	-0.59 (0.45)	0.42 (0.37)	0.47 (0.49)	0.35 (0.66)
Weidner	0.38 (0.41)	0.47 (0.58)	0.30 (0.57)	-0.12 (0.48)	-0.58 (0.70)	0.13 (0.79)	0.65 (0.56)	0.88 (0.76)	0.33 (0.88)
Vidal-Bralo	0.71 (0.27)	0.46 (0.43)	0.97 (0.33)	0.27 (0.36)	-0.50 (0.49)	0.68 (0.48)	0.98 (0.32)	0.82 (0.53)	1.20 (0.35)
DunedinPoAm	1.11 (0.01)	1.10 (0.01)	1.12 (0.01)	1.11 (0.01)	1.09 (0.01)	1.13 (0.01)	1.11 (0.01)	1.10 (0.01)	1.12 (0.01)

Abbreviations: DNAmAA, DNA-methylation age acceleration; DNAmAge, DNA-methylation age; SD, standard deviation.

eTable 6. Sensitivity analysis adjusting for all covariates listed in Table 1

	Overall participants		Participants with diabetes		Participants with pre-diabetes	
	HR (95% CI)	*P*	HR (95% CI)	*P*	HR (95% CI)	*P*
**Overall Survival**	662 deaths		347 deaths		315 deaths	
HorvathAge Accel	1.074(1.000,1.153)	0.051	1.046(0.945, 1.158)	0.385	1.099(0.987,1.223)	0.086
HannumAge Accel	1.068(0.991,1.152)	0.086	1.054(0.952, 1.167)	0.312	1.094(0.970,1.234)	0.142
SkinBloodAge Accel	0.999(0.924,1.081)	0.989	0.990(0.892, 1.100)	0.856	0.974(0.860,1.102)	0.673
**PhenoAge Accel**	**1.091(1.027,1.160)**	**0.005**	1.024(0.945, 1.110)	0.562	**1.179(1.071,1.296)**	**< 0.001**
**AgeAccelGrim2**	**1.339(1.208,1.484)**	**< 0.001**	**1.288(1.105, 1.500)**	**0.001**	**1.353(1.173, 1.561)**	**< 0.001**
ZhangAge Accel	1.115(0.889,1.399)	0.346	1.046(0.778, 1.405)	0.768	1.165(0.808,1.679)	0.414
LinAge Accel	1.021(0.970,1.075)	0.419	1.002(0.935, 1.074)	0.957	1.037(0.956,1.124)	0.383
WeidnerAge Accel	0.998(0.954,1.044)	0.934	0.995(0.929, 1.067)	0.896	1.025(0.961,1.094)	0.453
**Vidal-BraloAge Accel**	**1.086(1.002,1.177)**	**0.045**	1.067(0.953, 1.194)	0.261	1.120(0.994,1.262)	0.064
**DunedinPoAm**	**1.157(1.044,1.281)**	**0.005**	1.086(0.936, 1.259)	0.276	**1.181(1.021,1.366)**	**0.025**
**Cardiovascular Death**						
HorvathAge Accel	1.001(0.877, 1.143)	0.986	1.087(0.898, 1.316)	0.390	0.834(0.671, 1.036)	0.101
HannumAge Accel	1.051(0.912, 1.211)	0.490	1.042(0.866, 1.255)	0.662	1.083(0.848, 1.383)	0.523
SkinBloodAge Accel	1.005(0.868, 1.163)	0.948	1.037(0.847, 1.270)	0.725	0.875(0.699, 1.096)	0.246
PhenoAge Accel	1.086(0.967, 1.219)	0.163	1.067(0.915, 1.244)	0.411	1.143(0.943, 1.385)	0.173
**AgeAccelGrim2**	**1.490(1.226, 1.811)**	**< 0.001**	**1.386(1.051, 1.826)**	**0.021**	**1.575(1.200, 2.067)**	**0.001**
ZhangAge Accel	1.007(0.678, 1.497)	0.971	1.021(0.608, 1.716)	0.936	0.847(0.448, 1.601)	0.608
LinAge Accel	1.006(0.914, 1.106)	0.907	1.021(0.900, 1.157)	0.751	0.930(0.790, 1.095)	0.383
WeidnerAge Accel	1.009(0.927, 1.098)	0.841	1.002(0.878, 1.143)	0.979	1.013(0.892, 1.152)	0.839
Vidal-BraloAge Accel	0.986(0.848, 1.147)	0.858	1.009(0.823, 1.236)	0.935	0.901(0.709, 1.146)	0.901
**DunedinPoAm**	**1.248(1.030, 1.514)**	**0.024**	1.031(0.783, 1.357)	0.828	**1.390(1.056, 1.830**	**0.019**

**Non-Cardiovascular Death**						
**HorvathAge Accel**	**1.100(1.012, 1.197)**	**0.026**	1.029(0.911, 1.161)	0.647	**1.199(1.061,1.355)**	**0.004**
HannumAge Accel	1.074(0.983, 1.174)	0.115	1.058(0.936, 1.195)	0.369	1.099(0.957,1.263)	0.180
SkinBloodAge Accel	0.995(0.906, 1.092)	0.910	0.970(0.858, 1.098)	0.634	1.010(0.871,1.170)	0.896
**PhenoAge Accel**	**1.090(1.015, 1.171)**	**0.018**	1.006(0.914, 1.107)	0.898	**1.192(1.068, 1.331)**	**0.002**
**AgeAccelGrim2**	**1.283(1.137, 1.448)**	**< 0.001**	**1.232(1.025, 1.481)**	**0.026**	**1.295(1.094, 1.532)**	**0.003**
ZhangAge Accel	1.153(0.879, 1.514)	0.304	1.050(0.734, 1.503)	0.789	1.321(0.850,2.052)	0.215
LinAge Accel	1.026(0.966, 1.090)	0.397	0.994(0.915, 1.081)	0.889	1.078(0.981,1.184)	0.119
WeidnerAge Accel	0.992(0.940, 1.046)	0.758	0.991(0.913, 1.075)	0.821	1.027(0.952,1.107)	0.498
**Vidal-BraloAge Accel**	**1.125(1.023, 1.237)**	**0.015**	1.093(0.954, 1.252)	0.202	**1.209(1.051,1.391)**	**0.008**
DunedinPoAm	1.126(0.998, 1.270)	0.053	1.103(0.925, 1.316)	0.275	1.106(0.930,1.315)	0.256

Models were adjusted for each DNAmAA and all covariates listed in Table 1.
Bold indicates significant at $P < 0.05$.
Abbreviations: CI, confidence interval; DNAmAA, DNA-methylation age acceleration; HR, hazard ratio

eTable 7. Sensitivity analysis after excluding participants with missing variables

	Overall participants (n = 1000)		Participants with diabetes (n = 440)		Participants with pre-diabetes (n = 560)	
	HR (95% CI)	*P*	HR (95% CI)	*P*	HR (95% CI)	*P*
**Overall survival**						
HorvathAge Accel	1.058 (0.973, 1.150)	0.187	1.012 (0.899, 1.139)	0.846	1.064 (0.945, 1.198)	0.303
**HannumAge Accel**	**1.096 (1.001, 1.200)**	**0.047**	1.072 (0.944, 1.218)	0.285	1.084 (0.948, 1.240)	0.237
SkinBloodAge Accel	0.963 (0.887, 1.047)	0.379	0.932 (0.840, 1.035)	0.188	0.949 (0.830, 1.086)	0.446
**PhenoAge Accel**	**1.090 (1.016, 1.170)**	**0.016**	0.993 (0.901, 1.094)	0.887	**1.175 (1.061, 1.302)**	**0.002**
**AgeAccelGrim2**	**1.305 (1.167, 1.460)**	**< 0.001**	**1.246 (1.060, 1.464)**	**0.008**	**1.313 (1.121, 1.538)**	**< 0.001**
ZhangAge Accel	0.988 (0.79, 1.254)	0.920	0.893 (0.661, 1.207)	0.461	1.002 (0.680, 1.475)	0.993
LinAge Accel	1.025 (0.964, 1.090)	0.423	0.979 (0.900, 1.065)	0.628	1.046 (0.957, 1.144)	0.320
WeidnerAge Accel	1.008 (0.957, 1.062)	0.753	1.033 (0.958, 1.114)	0.401	1.005 (0.931, 1.084)	0.901
**Vidal-BraloAge Accel**	**1.137 (1.041, 1.242)**	**0.004**	1.114 (0.979, 1.266)	0.101	**1.166 (1.027, 1.324)**	**0.018**
DunedinPoAm	1.074 (0.965, 1.196)	0.191	1.004 (0.868, 1.161)	0.958	1.132 (0.963, 1.329)	0.132
**Cardiovascular mortality**						
HorvathAge Accel	0.940 (0.814, 1.086)	0.401	1.020 (0.822, 1.267)	0.854	0.770 (0.606, 0.977)	0.032
HannumAge Accel	1.115 (0.937, 1.326)	0.220	1.160 (0.915, 1.471)	0.221	0.997 (0.755, 1.316)	0.982
SkinBloodAge Accel	0.968 (0.831, 1.127)	0.672	1.011 (0.811, 1.260)	0.924	0.812 (0.630, 1.046)	0.107
**PhenoAge Accel**	1.077 (0.944, 1.230)	0.271	1.075 (0.893, 1.293)	0.447	1.075 (0.872, 1.326)	0.499
**AgeAccelGrim2**	**1.343 (1.090, 1.656)**	**0.006**	1.232 (0.925, 1.641)	0.153	**1.414 (1.044, 1.914)**	**0.025**
ZhangAge Accel	0.928 (0.613, 1.405)	0.724	1.035 (0.565, 1.896)	0.911	0.632 (0.316, 1.264)	0.194
LinAge Accel	1.016 (0.907, 1.137)	0.785	1.016 (0.870, 1.186)	0.841	0.941 (0.783, 1.131)	0.515
WeidnerAge Accel	1.021 (0.928, 1.124)	0.670	1.041 (0.906, 1.195)	0.574	1.044 (0.904, 1.205)	0.559
Vidal-BraloAge Accel	1.026 (0.870, 1.209)	0.764	1.056 (0.837, 1.331)	0.648	0.940 (0.726, 1.217)	0.640

**DunedinPoAm**	1.153 (0.948, 1.403)	0.153	0.882 (0.674, 1.154)	0.360	**1.378 (1.028, 1.846)**	**0.032**
**Non-cardiovascular mortality**						
**HorvathAge Accel**	**1.111 (1.006, 1.227)**	**0.039**	1.011 (0.876, 1.167)	0.877	**1.175 (1.026, 1.344)**	**0.020**
**HannumAge Accel**	1.088 (0.978, 1.210)	0.121	1.035 (0.890, 1.204)	0.655	1.111 (0.953, 1.296)	0.179
SkinBloodAge Accel	0.961 (0.871, 1.060)	0.431	0.906 (0.804, 1.022)	0.108	0.992 (0.847, 1.162)	0.923
**PhenoAge Accel**	**1.090 (1.005, 1.186)**	**0.039**	0.962 (0.858, 1.079)	0.510	**1.200 (1.067, 1.350)**	**0.002**
**AgeAccelGrim2**	**1.285 (1.126, 1.467)**	**< 0.001**	**1.256 (1.034, 1.526)**	**0.022**	**1.274 (1.058, 1.532)**	**0.010**
ZhangAge Accel	1.010 (0.758, 1.344)	0.947	0.844 (0.595, 1.198)	0.342	1.153 (0.725, 1.832)	0.548
LinAge Accel	1.030 (0.958, 1.107)	0.431	0.966 (0.873, 1.069)	0.502	1.078 (0.973, 1.194)	0.152
WeidnerAge Accel	1.000 (0.940, 1.064)	0.989	1.031 (0.942, 1.129)	0.507	0.989 (0.904, 1.082)	0.811
**Vidal-BraloAge Accel**	**1.184 (1.067, 1.314)**	**0.001**	1.142 (0.978, 1.333)	0.092	**1.257 (1.086, 1.454)**	**0.002**
DunedinPoAm	1.048 (0.922, 1.191)	0.477	1.060 (0.892, 1.259)	0.510	1.038 (0.857, 1.258)	0.704

Models were adjusted for chronological age, sex, ethnicity, poverty income ratio, smoking status, body mass index, geriatric nutritional risk index, atherosclerotic cardiovascular disease, hypertension, hyperlipidemia, and chronic kidney disease.
Bold indicates significant at $P < 0.05$.
Abbreviations: CI, confidence interval; HR, hazard ratio.

eTable 8. Sensitivity analysis after excluding participants with missing variables for associations between GrimAA tertiles and mortality outcomes

	Overall participants (N = 1000)		Participants with diabetes (N = 440)		Participants with pre-diabetes (N = 560)	
	HR (95% CI)	P	HR (95% CI)	P	HR (95% CI)	P
**Overall Survival**	528 deaths		275 deaths		253 deaths	
AgeAccelGrim2 T1	Reference	Reference	Reference	Reference	Reference	Reference
AgeAccelGrim2 T2	1.099 (0.875, 1.380)	0.419	1.063 (0.782, 1.445)	0.697	1.046 (0.744, 1.472)	0.795
AgeAccelGrim2 T3	1.553 (1.205, 2.002)	< 0.001	1.369 (0.972, 1.929)	0.073	1.505 (1.020, 2.220)	0.039
**Cardiovascular Death**	149 deaths		82 deaths		67 deaths	
AgeAccelGrim2 T1	Reference	Reference	Reference	Reference	Reference	Reference
AgeAccelGrim2 T2	1.024 (0.663, 1.580)	0.915	0.982 (0.559, 1.723)	0.949	1.066 (0.526, 2.158)	0.860
AgeAccelGrim2 T3	1.484 (0.921, 2.390)	0.105	1.428 (0.777, 2.623)	0.251	1.640 (0.743, 3.619)	0.221
**Non-Cardiovascular Death**	379 deaths		193 deaths		186 deaths	
AgeAccelGrim2 T1	Reference	Reference	Reference	Reference	Reference	Reference
AgeAccelGrim2 T2	1.122 (0.858, 1.468)	0.399	1.096 (0.759, 1.584)	0.625	1.045 (0.707, 1.545)	0.824
AgeAccelGrim2 T3	1.565 (1.159, 2.114)	0.003	1.354 (0.896, 2.048)	0.150	1.471 (0.940, 2.303)	0.091

Cox proportional hazards models were adjusted for chronological age, sex, ethnicity, poverty income ratio, smoking status, body mass index, geriatric nutritional risk index, cardiovascular disease, hypertension, hyperlipidemia, and chronic kidney disease.

Abbreviations: CI, confidence interval; HR, hazard ratio.

eTable 9. Sensitivity analysis after excluding participants who died within 2-year follow-up

	Overall participants (n = 1,155)		Participants with diabetes (n = 510)		Participants with pre-diabetes (n = 560)	
	HR (95% CI)	*P*	HR (95% CI)	*P*	HR (95% CI)	*P*
**Overall Survival**						
HorvathAge Accel	1.059 (0.983, 1.141)	0.129	0.992 (0.897, 1.097)	0.873	1.108 (0.996, 1.234)	0.060
HannumAge Accel	1.054 (0.975, 1.139)	0.185	1.002 (0.905, 1.099)	0.971	1.089 (0.969, 1.223)	0.153
SkinBloodAge Accel	0.983 (0.908, 1.065)	0.679	0.936 (0.851, 1.030)	0.177	1.012 (0.891, 1.150)	0.850
**PhenoAge Accel**	**1.095 (1.030, 1.165)**	**0.004**	1.012 (0.933, 1.097)	0.778	**1.194 (1.086, 1.313)**	**< 0.001**
**AgeAccelGrim2**	**1.309 (1.184, 1.447)**	**< 0.001**	**1.228 (1.064, 1.417)**	**0.005**	**1.338 (1.161, 1.543)**	**< 0.001**
ZhangAge Accel	1.070 (0.849, 1.348)	0.569	0.908 (0.692, 1.192)	0.488	1.264 (0.861, 1.855)	0.232
LinAge Accel	1.021 (0.969, 1.076)	0.441	0.982 (0.915, 1.054)	0.615	1.046 (0.966, 1.133)	0.270
WeidnerAge Accel	1.011 (0.965, 1.059)	0.650	0.996 (0.927, 1.069)	0.902	1.037 (0.973, 1.106)	0.265
**Vidal-BraloAge Accel**	1.083 (0.998, 1.175)	0.055	1.018 (0.908, 1.141)	0.760	**1.149 (1.022, 1.292)**	**0.020**
**DunedinPoAm**	**1.140 (1.036, 1.256)**	**0.008**	1.083 (0.949, 1.237)	0.236	**1.161 (1.009, 1.335)**	**0.037**
**Cardiovascular Death**						
HorvathAge Accel	0.968 (0.845, 1.109)	0.635	1.021 (0.850, 1.226)	0.824	0.824 (0.663, 1.025)	0.083
HannumAge Accel	0.999 (0.866, 1.153)	0.992	0.983 (0.822, 1.175)	0.853	0.953 (0.752, 1.208)	0.691
SkinBloodAge Accel	0.973 (0.841, 1.126)	0.711	0.958 (0.803, 1.142)	0.631	0.897 (0.705, 1.140)	0.373
PhenoAge Accel	1.086 (0.965, 1.223)	0.172	1.050 (0.905, 1.217)	0.522	1.098 (0.902, 1.337)	0.350
**AgeAccelGrim2**	**1.449 (1.198, 1.752)**	**< 0.001**	**1.328 (1.024, 1.721)**	**0.032**	**1.497 (1.134, 1.974)**	**0.004**
ZhangAge Accel	0.926 (0.624, 1.376)	0.705	0.877 (0.553, 1.391)	0.577	0.785 (0.403, 1.531)	0.478
LinAge Accel	1.000 (0.905, 1.105)	0.997	1.009 (0.889, 1.147)	0.887	0.920 (0.780, 1.086)	0.323
WeidnerAge Accel	1.024 (0.939, 1.117)	0.591	1.032 (0.908, 1.175)	0.625	1.033 (0.912, 1.170)	0.607
Vidal-BraloAge Accel	1.032 (0.886, 1.201)	0.689	1.034 (0.843, 1.268)	0.749	0.985 (0.777, 1.250)	0.904

**DunedinPoAm**	**1.215 (1.016, 1.452)**	**0.032**	1.069 (0.838, 1.363)	0.591	1.284 (0.988, 1.670)	0.062
**Non-Cardiovascular Death**						
**HorvathAge Accel**	**1.094 (1.003, 1.194)**	**0.043**	0.977 (0.866, 1.103)	0.712	**1.212 (1.074, 1.369)**	**0.002**
**HannumAge Accel**	1.075 (0.981, 1.178)	0.124	1.006 (0.889, 1.138)	0.928	1.136 (0.994, 1.299)	0.061
SkinBloodAge Accel	0.985 (0.896, 1.083)	0.755	0.924 (0.825, 1.035)	0.171	1.050 (0.904, 1.220)	0.521
**PhenoAge Accel**	**1.095 (1.018, 1.176)**	**0.014**	0.992 (0.901, 1.093)	0.876	**1.219 (1.094, 1.358)**	**< 0.001**
**AgeAccelGrim2**	**1.257 (1.117, 1.415)**	**< 0.001**	1.185 (0.998, 1.407)	0.052	**1.290 (1.093, 1.521)**	**0.003**
ZhangAge Accel	1.129 (0.853, 1.494)	0.396	0.915 (0.656, 1.276)	0.599	1.491 (0.944, 2.355)	0.087
LinAge Accel	1.028 (0.967, 1.093)	0.378	0.970 (0.890, 1.056)	0.481	1.086 (0.992, 1.190)	0.075
WeidnerAge Accel	1.003 (0.949, 1.061)	0.913	0.978 (0.898, 1.065)	0.604	1.036 (0.961, 1.116)	0.360
**Vidal-BraloAge Accel**	1.100 (1.000, 1.211)	0.051	1.008 (0.878, 1.157)	0.915	**1.208 (1.056, 1.383)**	**0.006**
DunedinPoAm	1.115 (0.995, 1.251)	0.061	1.093 (0.934, 1.280)	0.268	1.116 (0.946, 1.317)	0.192

Models were adjusted for chronological age, sex, ethnicity, poverty income ratio, smoking status, body mass index, geriatric nutritional risk index, atherosclerotic cardiovascular disease, hypertension, hyperlipidemia, and chronic kidney disease

Bold indicates significant at $P < 0.05$

Abbreviations: CI, confidence interval; HR, hazard ratio,

eTable 10. Sensitivity analysis after excluding participants who died within 2-year follow-up for associations between GrimAA tertiles and mortality outcomes

	Overall participants (N = 1155)		Participants with diabetes (N = 440)		Participants with pre-diabetes (N = 560)	
	HR (95% CI)	P	HR (95% CI)	P	HR (95% CI)	P
**Overall Survival**	618 deaths		315 deaths		303 deaths	
AgeAccelGrim2 T1	Reference	Reference	Reference	Reference	Reference	Reference
AgeAccelGrim2 T2	1.108 (0.896, 1.371)	0.343	0.937 (0.702, 1.251)	0.657	1.188 (0.869, 1.624)	0.280
AgeAccelGrim2 T3	1.509 (1.201, 1.895)	< 0.001	1.383 (1.017, 1.882)	0.039	1.582 (1.117, 2.240)	0.010
**Cardiovascular Death**	173 deaths		95 deaths		78 deaths	
AgeAccelGrim2 T1	Reference	Reference	Reference	Reference	Reference	Reference
AgeAccelGrim2 T2	1.080 (0.712, 1.636)	0.718	0.870 (0.507, 1.493)	0.614	1.213 (0.627, 2.345)	0.567
AgeAccelGrim2 T3	1.847 (1.204, 2.835)	0.005	1.625 (0.940, 2.812)	0.082	2.135 (1.059, 4.305)	0.034
**Non-Cardiovascular Death**	445 deaths		220 deaths		225 deaths	
AgeAccelGrim2 T1	Reference	Reference	Reference	Reference	Reference	Reference
AgeAccelGrim2 T2	1.116 (0.871, 1.429)	0.386	0.964 (0.684, 1.358)	0.834	1.182 (0.829, 1.685)	0.356
AgeAccelGrim2 T3	1.390 (1.061, 1.821)	0.017	1.295 (0.892, 1.878)	0.174	1.440 (0.963, 2.152)	0.075

Cox proportional hazards models were adjusted for chronological age, sex, ethnicity, poverty income ratio, smoking status, body mass index, geriatric nutritional risk index, cardiovascular disease, hypertension, hyperlipidemia, and chronic kidney disease.

Abbreviations: CI, confidence interval; GrimAA, Grim age acceleration; HR, hazard ratio.

eTable 11. Associations between DNAmAA and mortality outcomes among general participants without diabetes/prediabetes

	Participants without diabetes/prediabetes or major chronic disease (N = 672)[a]		Participants without diabetes/prediabetes (N = 984)[b]	
	**HR (95% CI)**	***P***		
**Overall Survival**				
HorvathAge Accel	1.104(0.954,1.277)	0.184	1.014(0.919,1.119)	0.779
HannumAge Accel	1.067(0.932,1.223)	0.347	1.044(0.954,1.143)	0.344
SkinBloodAge Accel	0.996(0.859,1.156)	0.960	0.967(0.878,1.064)	0.489
**PhenoAge Accel**	**1.143(1.015,1.289)**	**0.028**	1.073(0.993,1.159)	0.075
**AgeAccelGrim2**	**1.386(1.156,1.661)**	**< 0.001**	**1.361(1.202,1.542)**	**< 0.001**
ZhangAge Accel	1.050(0.697,1.583)	0.815	0.918(0.713,1.182)	0.508
LinAge Accel	0.994(0.907,1.089)	0.897	0.994(0.936,1.054)	0.831
WeidnerAge Accel	0.991(0.913,1.076)	0.834	0.981(0.931,1.034)	0.479
Vidal-BraloAge **Accel**	0.959(0.829,1.109)	0.572	0.985(0.894,1.085)	0.757
**DunedinPoAm**	**1.467(1.200,1.793)**	**< 0.001**	**1.267(1.113,1.444)**	**< 0.001**
**Cardiovascular Death**				
HorvathAge Accel	1.165(0.855,1.588)	0.333	1.048(0.867,1.266)	0.629
HannumAge Accel	1.019(0.767,1.354)	0.896	1.157(0.972,1.377)	0.100
SkinBloodAge Accel	1.303(0.922,1.841)	0.133	1.156(0.929,1.439)	0.193
PhenoA**ge Accel**	1.176(0.918,1.507)	0.200	1.059(0.918,1.222)	0.430
**AgeAccelGrim2**	**1.494(1.025,2.178)**	**0.037**	**1.514(1.202,1.908)**	**< 0.001**
ZhangAge Accel	1.744(0.667,4.564)	0.257	1.206(0.693,2.099)	0.507
LinAge Accel	1.118(0.930,1.344)	0.236	1.064(0.952,1.190)	0.275
WeidnerAge Accel	1.044(0.886,1.231)	0.607	1.018(0.926,1.119)	0.709
Vidal-BraloAge Accel	1.107(0.827,1.481)	0.494	1.071(0.897,1.278)	0.451
**DunedinPoAm**	1.271(0.831,1.943)	0.268	**1.297(1.021,1.648)**	**0.033**
**Non-Cardiovascular Death**				
HorvathAge Accel	1.085(0.919,1.280)	0.336	1.000(0.891,1.123)	0.994
HannumAge Accel	1.084(0.928,1.266)	0.310	1.006(0.905,1.118)	0.915
SkinBloodAge Accel	0.934(0.795,1.097)	0.408	0.913(0.822,1.013)	0.087
PhenoAge Accel	1.135(0.990,1.300)	0.070	1.081(0.986,1.186)	0.099
**AgeAccelGrim2**	**1.358(1.105,1.669)**	**0.004**	**1.313(1.131,1.523)**	**< 0.001**
ZhangAge Accel	0.926(0.592,1.449)	0.737	0.832(0.627,1.104)	0.203
LinAge Accel	0.959(0.864,1.063)	0.426	0.967(0.901,1.037)	0.342
WeidnerAge Accel	0.975(0.888,1.071)	0.598	0.966(0.907,1.029)	0.282
Vidal-BraloAge Accel	0.914(0.772,1.082)	0.295	0.954(0.849,1.071)	0.423
**DunedinPoAm**	**1.533(1.220,1.927)**	**< 0.001**	**1.263(1.082,1.474)**	**0.003**

[a] Participants with diabetes, prediabetes, cancer, cardiovascular disease, chronic kidney disease were excluded from analysis, and Cox proportional hazards models were adjusted for chronological age, sex, ethnicity, poverty income ratio, smoking status, body mass index, geriatric nutritional risk index, hypertension and hyperlipidemia.

[b] Participants with diabetes, prediabetes, and cancer were excluded from analysis, and Cox proportional hazards models were adjusted for chronological age, sex, ethnicity, poverty income ratio, smoking status, body mass index, geriatric nutritional risk index, cardiovascular disease, hypertension, hyperlipidemia, and chronic kidney disease.

Bold indicates significant at $P < 0.05$.

Abbreviations: CI, confidence interval; DNAmAA, DNA-methylation age acceleration; HR, hazard ratio.

eTable 12. Subgroup analysis for associations between AgeAccelGrim2 and mortality outcomes in diabetes participants

	All-cause mortality		
Subgroup category	HR (95% CI)	*P*	*P-for-interaction*
Chronological age			
< 65 years	1.250 (1.004, 1.557)	0.046	0.981
≥ 65 years	1.350 (1.128, 1.614)	0.001	
Sex			
Male	1.255 (1.053, 1.497)	0.011	0.709
Female	1.415 (1.132, 1.768)	0.002	
BMI			
< 30 kg/m^2	1.222 (1.014, 1.474)	0.035	0.787
≥ 30 kg/m^2	1.400 (1.133, 1.729)	0.002	
	Cardiovascular mortality		
Subgroup category	HR (95% CI)	*P*	*P-for-interaction*
Chronological age			
< 65 years	1.377 (0.911, 2.082)	0.129	0.505
≥ 65 years	1.433 (1.037, 1.980)	0.029	
Sex			
Male	1.498 (1.094, 2.051)	0.012	0.697
Female	1.149 (0.741, 1.781)	0.535	
BMI			
< 30 kg/m^2	1.263 (0.882, 1.809)	0.203	0.322
≥ 30 kg/m^2	1.518 (1.041, 2.214)	0.030	
	Non-cardiovascular mortality		
Subgroup category	HR (95% CI)	*P*	*P-for-interaction*
Chronological age			
< 65 years	1.199 (0.925, 1.555)	0.171	0.697
≥ 65 years	1.297 (1.046, 1.608)	0.018	
Sex			
Male	1.136 (0.917, 1.406)	0.243	0.479
Female	1.537 (1.182, 1.998)	0.001	
BMI			
< 30 kg/m^2	1.204 (0.965, 1.502)	0.099	0.773
≥ 30 kg/m^2	1.334 (1.033, 1.723)	0.027	

Abbreviations: BMI, body mass index; CI, confidence interval; HR, hazard ratio.

eTable 13. Subgroup analysis for associations between AgeAccelGrim2 and mortality outcomes in pre-diabetes participants

	All-cause mortality		
Subgroup category	HR (95% CI)	*P*	*P-for-interaction*
Chronological age			
< 65 years	1.215 (0.946, 1.559)	0.127	0.602
≥ 65 years	1.417 (1.198, 1.675)	< 0.001	
Sex			
Male	1.366 (1.140, 1.638)	< 0.001	0.620
Female	1.349 (1.067, 1.706)	0.012	
BMI			
< 30 kg/m^2	1.310 (1.109, 1.547)	0.001	0.647
≥ 30 kg/m^2	1.340 (1.023, 1.756)	0.034	
	Cardiovascular mortality		
Subgroup category	HR (95% CI)	*P*	*P-for-interaction*
Chronological age			
< 65 years	1.496 (0.827, 2.706)	0.183	0.447
≥ 65 years	1.501 (1.106, 2.036)	0.009	
Sex			
Male	1.551 (1.088, 2.210)	0.015	0.624
Female	1.517 (0.961, 2.396)	0.073	
BMI			
< 30 kg/m^2	1.463 (1.069, 2.001)	0.017	0.555
≥ 30 kg/m^2	1.431 (0.806, 2.542)	0.221	
	Non-cardiovascular mortality		
Subgroup category	HR (95% CI)	*P*	*P-for-interaction*
Chronological age			
< 65 years	1.160 (0.879, 1.530)	0.295	0.789
≥ 65 years	1.385 (1.134, 1.692)	0.001	
Sex			
Male	1.323 (1.072, 1.633)	0.009	0.400
Female	1.293 (0.983, 1.701)	0.066	
BMI			
< 30 kg/m^2	1.262 (1.037, 1.536)	0.020	0.885
≥ 30 kg/m^2	1.317 (0.969, 1.791)	0.079	

Abbreviations: BMI, body mass index; CI, confidence interval; HR, hazard ratio.

eTable 14. Mediated effects of all mediators on the association AgeAccelGrim2 and all-cause mortality among participants with diabetes and pre-diabetes

Mediators	Average causal mediation effect		Average direct effect		Total effect		Proportion mediated	
	β (95% CI)	P	β (95% CI)	P	β (95% CI)	P	β (95% CI)	P
**Participants with diabetes**								
Body mass index, kg/m^2	0.001 (0.000, 0.000)	0.060	0.010 (0.006, 0.010)	< 0.001	0.011 (0.008, 0.010)	< 0.001	0.092 (0.004, 0.200)	0.060
Systolic blood pressure, mmHg	0.000 (-0.001, 0.000)	0.180	0.001 (-0.001, 0.000)	0.420	0.001 (-0.001, 0.000)	0.600	-0.428 (-1.597, 2.990)	0.780
Diastolic blood pressure, mmHg	0.000 (-0.001, 0.000)	0.140	-0.002 (-0.002, 0.000)	0.100	-0.002 (-0.002, 0.000)	0.060	0.165 (-0.215, 0.760)	0.200
**Hemoglobin A1c, %**	**0.005 (0.002, 0.010)**	**< 0.001**	**0.022 (0.008, 0.040)**	**0.020**	**0.027 (0.013, 0.040)**	**< 0.001**	**0.183 (0.074, 0.460)**	**< 0.001**
Total cholesterol, mmol/L	0.002 (-0.004, 0.010)	0.480	0.017 (-0.009, 0.040)	0.340	0.019 (-0.009, 0.040)	0.260	0.101 (-0.489, 1.510)	0.380
HDL-C, mmol/L	-0.009 (-0.031, 0.010)	0.320	0.024 (-0.080, 0.160)	0.620	0.015 (-0.088, 0.140)	0.740	-0.604 (-1.267, 2.740)	0.860
**eGFR, ml/min/1.73m^2**	**0.000 (-0.001, 0.000)**	**< 0.001**	**-0.002 (-0.002, 0.000)**	**0.004**	**-0.002 (-0.002, 0.000)**	**< 0.001**	**0.144 (0.038, 0.350)**	**< 0.001**
UACR, mg/g	0.000 (0.000, 0.000)	0.460	0.000 (0.000, 0.000)	< 0.001	0.000 (0.000, 0.000)	< 0.001	0.051 (-0.075, 0.320)	0.460
Systemic immune inflammation index	0.000 (0.000, 0.000)	< 0.001	0.000 (0.000, 0.000)	0.600	0.000 (0.000, 0.000)	0.440	1.903 (-7.062, 18.000)	0.440
C-reactive protein, mg/dl	0.008 (0.004, 0.010)	< 0.001	-0.002 (-0.021, 0.090)	0.860	0.006 (-0.013, 0.100)	0.820	1.303 (-4.274, 10.060)	0.820
**Oxidative Balance Score**	**-0.001 (-0.003, 0.000)**	**< 0.001**	**-0.006 (-0.008, 0.000)**	**0.020**	**-0.007 (-0.009, 0.000)**	**< 0.001**	**0.194 (0.103, 0.510)**	**< 0.001**
**Life's Simple 7 score**	**-0.007 (-0.012, 0.000)**	**< 0.001**	**-0.022 (-0.031, -0.010)**	**< 0.001**	**-0.029 (-0.034, -0.020)**	**< 0.001**	**0.234 (0.076, 0.580)**	**< 0.001**
**Frailty score**	**0.104 (0.044, 0.190)**	**< 0.001**	**0.313 (-0.076, 0.460)**	**0.040**	**0.416 (0.178, 0.530)**	**< 0.001**	**0.249 (0.100, 0.620)**	**< 0.001**
**Participants with pre-diabetes**								
Body mass index, kg/m^2	0.0001 (-0.001, 0.001)	0.280	0.003 (-0.002, 0.010)	0.200	0.004 (-0.001, 0.010)	0.160	0.146 (-1.006, 0.940)	0.360
Systolic blood pressure, mmHg	0.000 (-0.001, 0.000)	0.420	0.001 (-0.001, 0.000)	0.400	0.001 (-0.001, 0.000)	0.500	-0.210 (-1.476, 2.280)	0.840
Diastolic blood pressure, mmHg	-0.001 (-0.001, 0.000)	0.008	-0.001 (-0.003, 0.000)	0.540	-0.001 (-0.003, 0.000)	0.380	0.465 (-1.523, 1.940)	0.340
Hemoglobin A1c, %	0.024 (-0.001, 0.050)	0.060	-0.037 (-0.074, 0.040)	0.600	-0.013 (-0.049, 0.040)	0.860	-1.811 (-4.030, 4.650)	0.880
Total cholesterol, mmol/L	0.001 (-0.005, 0.010)	0.800	-0.028 (-0.047, 0.000)	0.060	-0.027 (-0.046, 0.000)	0.080	-0.023 (-1.130, 0.830)	0.880
HDL-cholesterol, mmol/L	-0.004 (-0.022, 0.020)	0.680	-0.020 (-0.120, 0.050)	0.580	-0.024 (-0.123, 0.060)	0.500	0.159 (-4.016, 1.820)	0.780

eGFR, ml/min/1.73m^2	0.000 (-0.001, 0.000)	0.200	-0.001 (-0.003, 0.000)	0.270	-0.002 (-0.003, 0.000)	0.200	0.165 (-1.293, 1.150)	0.380
UACR, mg/g	0.000 (0.000, 0.000)	0.520	0.001 (0.000, 0.001)	< 0.001	0.001 (0.000, 0.001)	< 0.001	0.027 (-0.049, 0.150)	0.520
Systemic immune inflammation index	0.000 (0.000, 0.000)	< 0.001	0.000 (0.000, 0.000)	0.120	0.000 (0.000, 0.000)	0.820	9.231 (-14.703, 18.200)	0.880
C-reactive protein, mg/dl	0.028 (0.016, 0.050)	< 0.001	0.013 (-0.014, 0.050)	0.420	0.040 (-0.007, 0.080)	0.020	0.687 (0.331, 2.500)	0.020
**Oxidative Balance Score**	**-0.002 (-0.004, 0.000)**	**< 0.001**	**-0.006 (-0.010, 0.000)**	**0.020**	**-0.009 (-0.012, 0.000)**	**< 0.001**	**0.277 (0.149, 0.660)**	**< 0.001**
**Life's Simple 7 score**	**-0.013 (-0.018, -0.010)**	**< 0.001**	**-0.013 (-0.024, 0.000)**	**0.120**	**-0.025 (-0.036, -0.010)**	**< 0.001**	**0.498 (0.274, 1.280)**	**< 0.001**
**Frailty score**	**0.074 (0.039, 0.150)**	**< 0.001**	**0.460 (0.129, 0.590)**	**< 0.001**	**0.534 (0.224, 0.640)**	**< 0.001**	**0.139 (0.067, 0.460)**	**< 0.001**

Abbreviations: CI, confidence interval; eGFR, estimated glomerular filtration rate; HDL-C, high-density lipoprotein cholesterol; UACR, urine albumin to creatinine ratio.

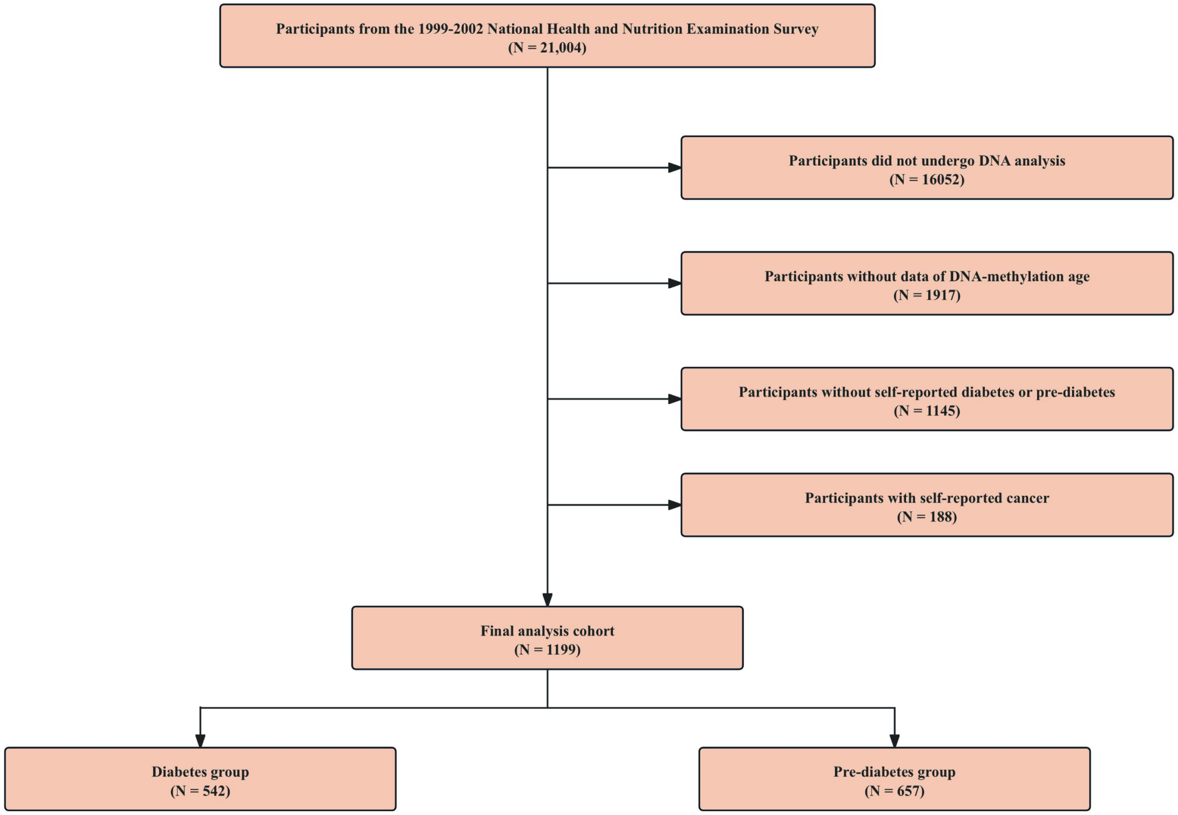

eFigure 1. Flowchart of this study

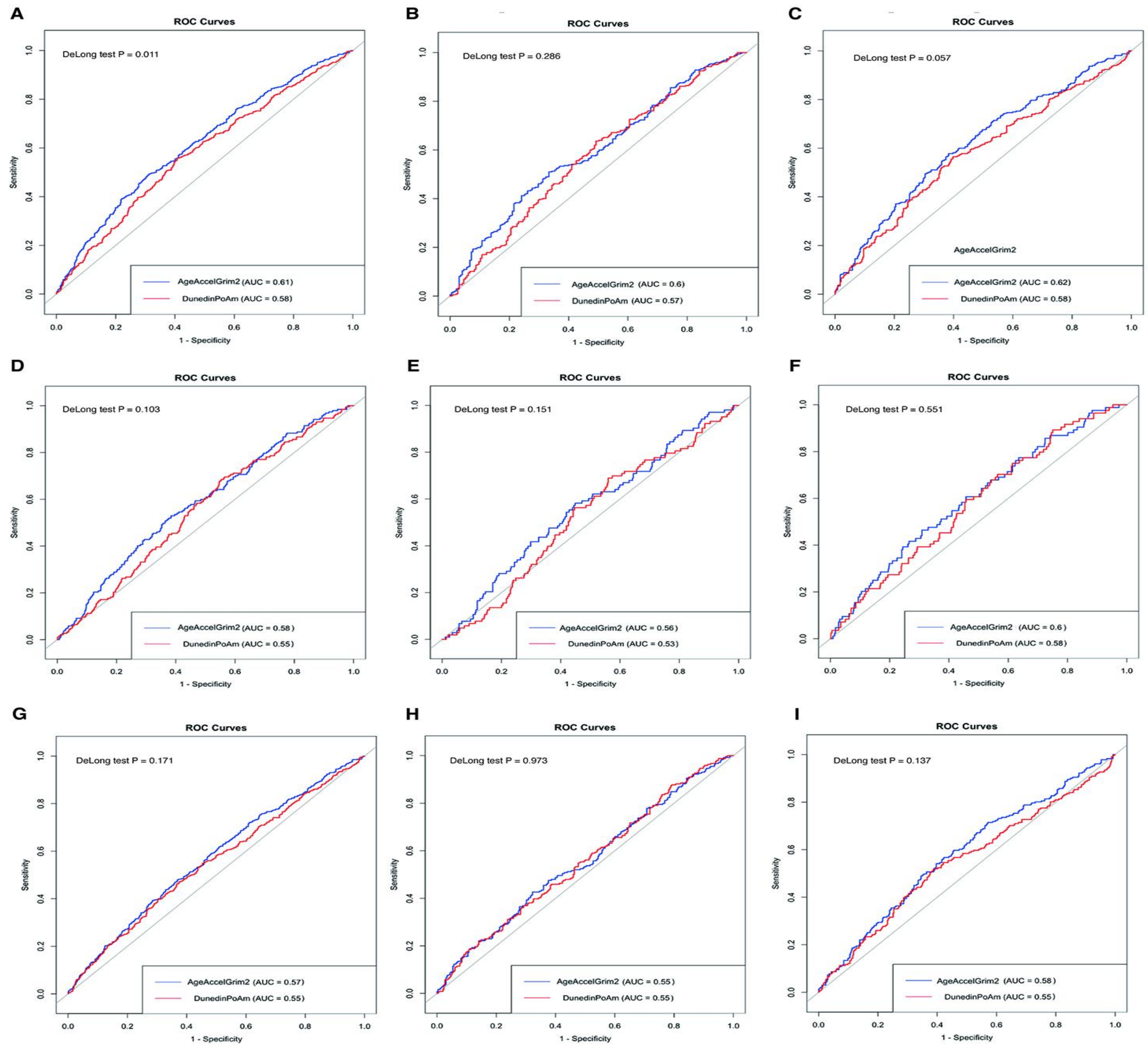

eFigure 2. Comparison the predictive performance of AgeAccelGrim2 and DunedinPoAm for mortality outcomes using ROC curve analysis

A

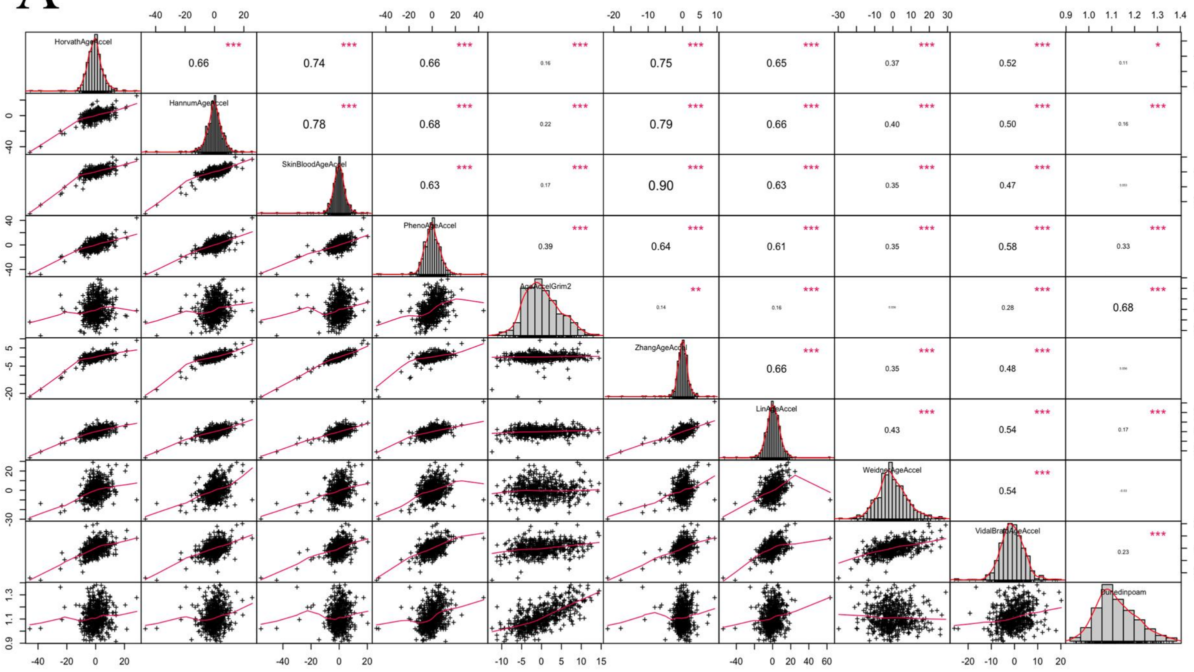

B

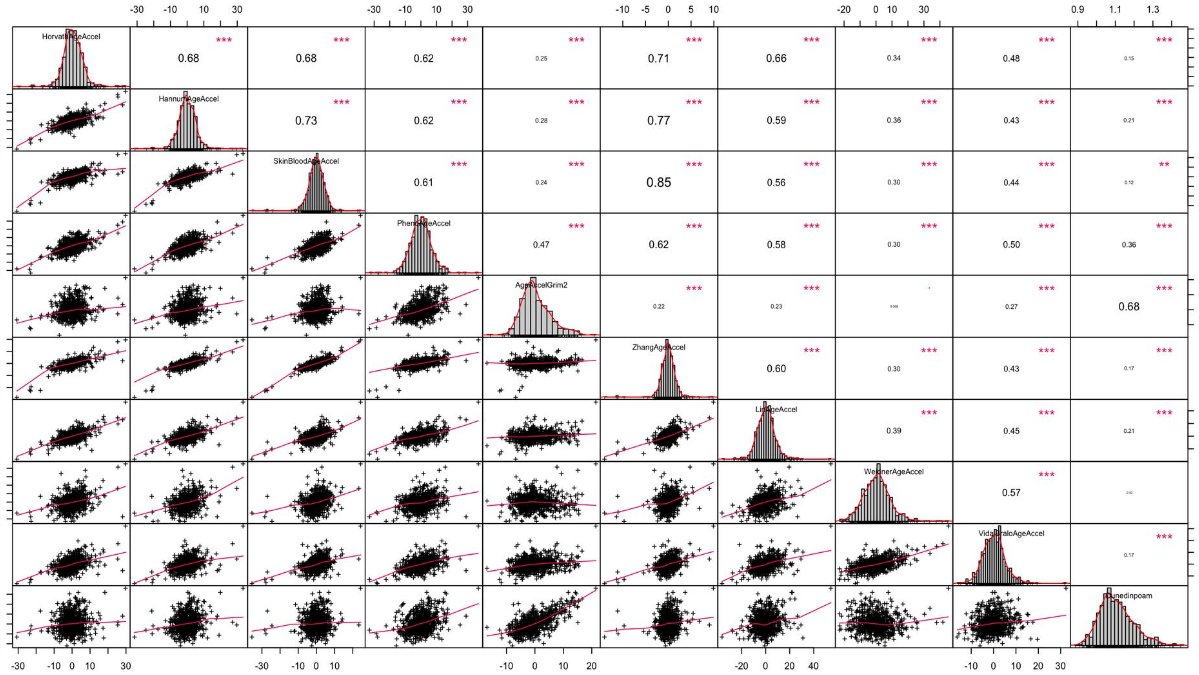

eFigure 3. The correlation analyses between each DNA-methylation age acceleration in populations with diabetes (A) and pre-diabetes (B). * P < 0.05; ** P < 0.01; *** P < 0.001. AA = age acceleration.

A

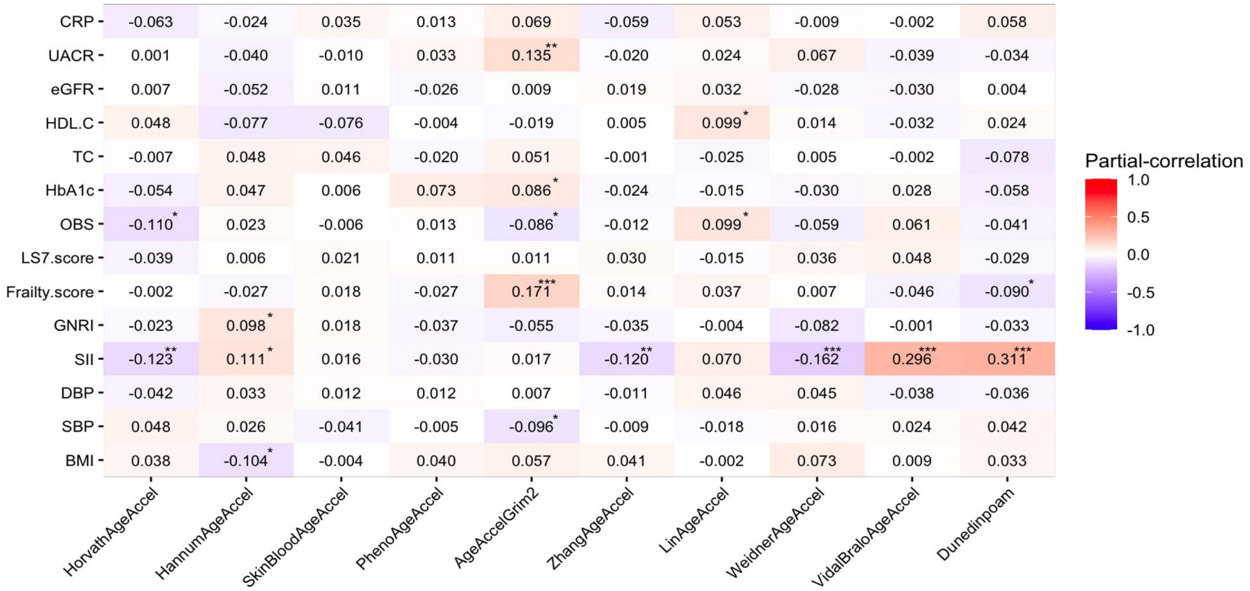

B

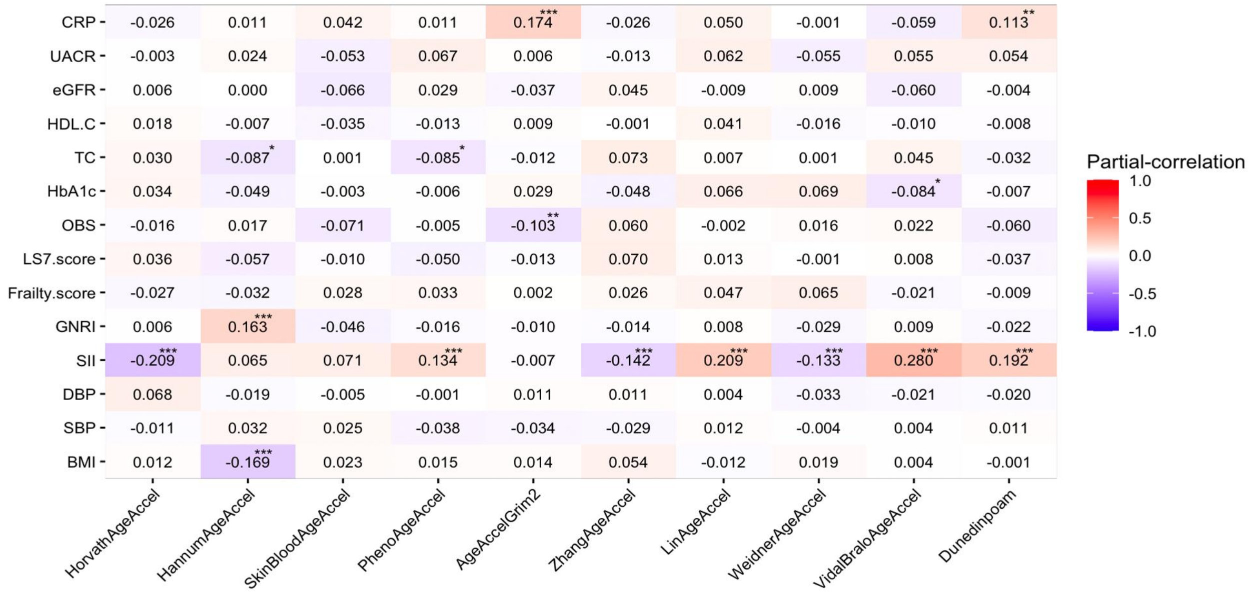

eFigure 4. Partial correlation analyses between different DNA-methylation age accelerations and multiple variables in populations with diabetes (A) and pre-diabetes (B). AA, age acceleration; BMI, body mass index; CRP, C-reactive protein; DBP, diastolic blood pressure; eGFR, estimated glomerular filtration rate; GNRI, Geriatric Nutritional Risk Index; HbA1c, hemoglobin A1c; HDL-C, high-density lipoprotein cholesterol; LS7, Life's Simple 7; SBP, systolic blood pressure; SII, Systemic Immune-inflammation Index; TC, total cholesterol; OBS, Oxidative Balance Score; UACR, urine albumin to creatinine ratio.
